# Prepregnancy Obesity Reprograms Offspring Skeletal Muscle Fibre Transition Through H3K9me3

**DOI:** 10.1002/jcsm.13825

**Published:** 2025-04-21

**Authors:** Yichi Wu, Sujuan Li, Jingyi Zhang, Anran Tian, Xiangyao Wang, Xi Yang, Fucheng Meng, Qing Li, Yuan Gao, Yingying Li, Furong Liang, Minglan Yao, Xiaoping Luo, Cai Zhang

**Affiliations:** ^1^ Department of Pediatrics Tongji Hospital, Tongji Medical College Huazhong University of Science and Technology Wuhan Hubei China; ^2^ Hubei Key Laboratory of Pediatric Genetic Metabolic and Endocrine Rare Diseases Wuhan China; ^3^ Department of Stomatology Tongji Hospital, Tongji Medical College Huazhong University of Science and Technology Wuhan China

**Keywords:** epigenetic, insulin resistance, muscle fibre type, prepregnancy obesity, skeletal muscle

## Abstract

**Background:**

Maternal prepregnancy obesity predisposes offspring to obesity and metabolic disorders, yet its impact on skeletal muscle fibre transition remains unclear. Given that skeletal muscle plays a crucial role in systemic metabolism, we investigated how maternal prepregnancy high‐fat diet (HFD) influences muscle fibre composition and metabolic function in offspring.

**Methods:**

We established mouse models with a prepregnancy chow diet (CD) and a prepregnancy high‐fat diet (HFD) for 8 weeks to compare metabolic phenotypes in offspring. Skeletal muscles from offspring were analysed using RNA sequencing, quantitative reverse transcription polymerase chain reaction and western blot to understand the changes in metabolic and signalling pathways. siRNA knockdown and lentiviral‐mediated overexpression experiments were conducted in vitro and in vivo to validate molecular mechanisms. Chromatin immunoprecipitation followed by qPCR (ChIP‐qPCR) was used to assess histone modification levels at promoter regions.

**Results:**

Male and female offspring of prepregnancy obese dams (mHFD) exhibited a significant reduction in slow‐twitch oxidative fibres (*p* < 0.001) and an increase in fast‐twitch glycolytic fibres compared with controls. This was accompanied by impaired glucose tolerance (AUC increased by 12.87%, *p* < 0.01), insulin resistance and mitochondrial dysfunction (mtDNA copy number reduced by 31%, *p* < 0.01). RNA sequencing identified IDH2 as the most significantly downregulated gene (29.67% decrease, *p* < 0.001), with protein levels further reduced in male (30.15%, *p* < 0.01) and female (46.02%, *p* < 0.0001) offspring. IDH2 knockdown in C2C12 cells impaired mitochondrial biogenesis and led to higher oxidative stress (NADP+/NADPH ratio elevated by 32%, *p* < 0.01), while IDH2 overexpression restored mitochondrial integrity, enhanced slow‐twitch fibre proportion (26.43 ± 0.6936% in mHFD‐LV‐IDH2, *p* < 0.01) and improved glucose metabolism (fasting glucose reduced by 14.7%, *p* < 0.01). ChIP‐qPCR revealed increased H3K9me3 enrichment at the IDH2 promoter (2.54‐fold in males, 2.55‐fold in females, *p* < 0.0001), suggesting transgenerational epigenetic regulation.

**Conclusions:**

Maternal prepregnancy obesity induces a metabolic shift in offspring skeletal muscle by promoting a slow‐to‐fast fibre transition and impairing mitochondrial biogenesis. This effect is mediated by IDH2 suppression via H3K9me3 histone modification, contributing to systemic insulin resistance. Targeting IDH2 may represent a potential therapeutic strategy to mitigate metabolic dysfunction in offspring exposed to maternal prepregnancy obesity.

## 1 Introduction

The prevalence of obesity has increased substantially among women of reproductive age worldwide over the past two decades [1, 2]. Maternal overnutrition is a pivotal factor that shapes the developmental environment in early life and increases the risk of metabolic diseases in offspring, such as obesity and diabetes [3, 4, 5, 6, 7, 8, 9, 10, 11, 12]. Maternal obesity, including prepregnancy obesity and excessive gestational weight gain (EGWG), impacts offspring metabolism differently. Prepregnancy obesity is more prevalent globally and affects early embryo development, whereas EGWG affects mid‐to‐late pregnancy. While gestational obesity has been extensively studied, fewer studies have focused on prepregnancy obesity.

Skeletal muscle is recognised as a key organ in energy metabolism. As the most abundant tissue in the human body, accounting for approximately 30%–40% of body mass, it serves as the primary site for insulin‐mediated glucose uptake [13, 14]. The capacity of skeletal muscle to perform various functions, such as locomotion, thermogenesis and metabolism, is predominantly ascribed to the adaptability of muscle fibres [15]. Muscle fibres are primarily classified into three types based on different contraction speeds and metabolic activity: slow‐twitch oxidative fibres, fast‐twitch oxidative fibres and fast‐twitch glycolytic fibres [16]. It has been well‐documented that individuals with obesity and T2D exhibit a shift in muscle fibre composition, characterised by a reduction in oxidative Type I fibres and an increase in glycolytic fibres, which is closely linked to insulin resistance and mitochondrial dysfunction [17, 18, 19]. However, until now, there are no studies reporting muscle fibre type transition or skeletal muscle metabolism in offspring of mothers with prepregnancy obesity.

Epigenetic modifications have garnered significant attention in the metabolic diseases [12, 20, 21, 22]. Histone modifications, in particular, are believed to play a key role in the developmental programming of offspring in response to environmental exposures, which affect gene expression levels by regulating chromatin accessibility without altering the genomic sequence [21, 22]. Importantly, recent studies have highlighted the crucial roles of histone modifications in maintaining skeletal muscle function and regulating muscle fibre types [23, 24]. While no studies to date have explored the regulation of histone modifications in the muscles of offspring exposed to an adverse intrauterine environment, it is plausible to speculate that histone modifications may modulate muscle function and metabolism in offspring subjected to intrauterine overnutrition.

Therefore, clarifying the effects of prepregnancy obesity on offspring and the underlying mechanisms will help to develop novel preventive and therapeutic strategies. In this study, we constructed mouse models with prepregnancy chow diet (CD) and prepregnancy HFD to compare the metabolic phenotypes and muscle fibre types in the offspring of two groups and to investigate the potential role of epigenetic regulation in this process.

## 2 Methods

### 2.1 Animals

Four‐week‐old C57BL/6J mice were purchased from GemPharmatech Co. Ltd. (Jiangsu, China). Female mice were given either a standard diet or a high‐fat diet (60%) before pregnancy, followed by a standard diet during pregnancy and lactation. Male mice used for breeding were continuously maintained on a chow diet. After 8 weeks of feeding, pregnancy was determined by the presence of a vaginal plug and assigned the embryonic age E0.5. All offspring were weaned onto a standard CD postnatally. Animals were anaesthetised with 1% pentobarbital before tissue collection. Samples were harvested at embryonic day 18.5 (E18.5) and at 8 weeks of age in offspring. Immediately after collection, tissues were flash‐frozen in liquid nitrogen and stored at −80°C for further analysis. All animal procedures complied with the guidelines and regulations of the Chinese Laboratory Animal Guidelines and were approved by the Animal Care and Use Committee of Tongji Hospital (Approval No.: TJH‐202209006).

### 2.2 Grip Strength Measurement and Treadmill Running

Grip strength was assessed in 8‐week‐old mice using a digital grip strength meter (BIOSEB, Vitrolles, France). To perform the test, the mice were placed on a grid with their bodies horizontal and their tails were gently pulled to determine the peak force, which was normalised by lean body mass. Each mouse was subjected to three trials at intervals greater than 15 min.

Mice were tested for exercise tolerance using a treadmill (Jeung Do Bio & Plant). To acclimatise the mice to the treadmill, they were run at 10 m/min for 5 min per day for 3 days. The mice were then tested by running on Day 4. The treadmill was run at 10 m/min for the first 5 min and then increased by 2 m/min every 2 min to 46 m/min until exhaustion. The running time of each mouse was recorded. Exhaustion was defined as the animal's inability to run on the treadmill for 10 s despite mechanical stimulation.

### 2.3 Additional Methods

Additional details regarding the methods and materials are provided in the Supporting Information.

### 2.4 Statistical Analysis

Data are presented as mean ± SEM. Statistical analyses and graphical presentations were performed using the GraphPad Prism 9.1 software. Normality and homogeneity of variance were tested before applying parametric tests. Two‐group comparisons were conducted using an unpaired Student's *t*‐test, while multiple group comparisons were performed using one‐way ANOVA followed by Dunnett's post‐hoc test. Significant differences are indicated as *p <* 0.05.

## 3 Results

### 3.1 Maternal Prepregnancy Obesity Induces Systemic and Muscular Insulin Resistance in Offspring

After 8 weeks of HFD feeding (60% calorie from fat), the maternal mice developed obesity, evidenced by a greater than 20% increase in body weight. Additionally, they developed hyperlipidaemia, higher hepatic lipid content, as well as elevated fasting blood glucose and insulin levels (Supplementary Figure 1A–E). The litter sizes and survival rates of offspring from different groups were comparable (Supplementary Figure 2A,B).

**FIGURE 1:**
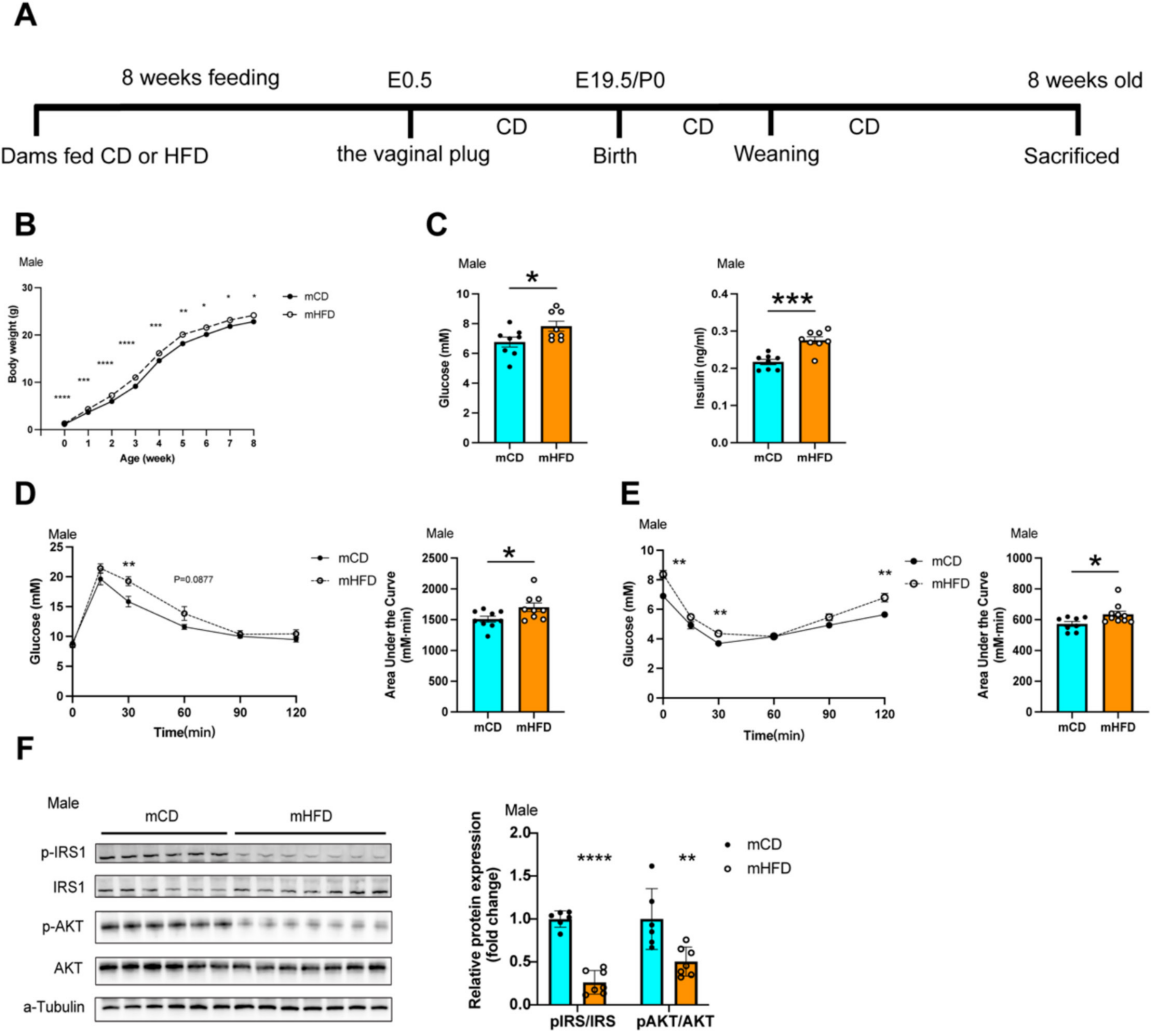
Maternal prepregnancy obesity induces glucose intolerance and altered insulin signalling in the male offspring. (A) Schematic representation of the animal model. Female mice were fed either a high‐fat diet (HFD; 60% calories from fat) or a standard chow diet (CON; 10% calories from fat) for 8 weeks before mating. (B–F) Metabolic parameters were measured in mCD and mHFD male offspring (*n* = 8). (B) Body weight and (C) serum fasting glucose levels and serum insulin levels were assessed. (D) Offspring underwent a glucose tolerance test (GTT) after 8 h of fasting and (E) an insulin tolerance test (ITT) after 6 h of fasting. (F) Western blot analysis of tyrosine phosphorylation of IRS1 (Ser636) and serinephosphorylation of AKT (Ser473) in male offspring muscle (*n* = 6–7). Data are presented as mean ± SEM. Unpaired Student's *t*‐test with two‐tailed distribution was used in data analyses. *p < 0.05, **p < 0.01, ***p < 0.001 and ****p < 0.0001.

In the offspring, both male and female of mothers with prepregnancy obesity (mHFD) had significantly increased birth weights compared with those of mothers with a prepregnancy chow diet (mCD). These weight differences persisted until weaning but declined in female offspring during subsequent growth (Figure 1B and Supplementary Figure 3A). At 8 weeks of age, although no weight differences were observed in females, fasting glucose and fasting insulin were significantly elevated in both male and female mHFD (Figure 1C and Supplementary Figure 3B,C). Consistently, mHFD exhibited significantly higher blood glucose levels at 30 min (19.27 ± 0.74 vs. 15.84 ± 0.89 mmol/L, *p <* 0.01) and 60 min (13.84 ± 1.15 vs. 11.61 ± 0.42 mmol/L, *p* = 0.087) during the glucose tolerance test (GTT) compared with mCD (Figure 1D). Furthermore, the area under the curve (AUC) for glucose tolerance was 12.87% higher in mHFD (*p <* 0.01) (Figure 1D). During the insulin tolerance test (ITT), mHFD exhibited a slower decline in blood glucose levels, with levels remaining significantly higher at 30 min (4.36 ± 0.09 vs. 3.69 ± 0.21 mmol/L, *p <* 0.01) and 120 min (6.79 ± 0.29 vs. 5.64 ± 0.14 mmol/L, *p <* 0.01) compared with mCD offspring (Figure 1E). Similar results were observed in female offspring (Supplementary Figure 3D,E). These results indicate that mHFD exhibited impaired glucose tolerance and insulin resistance.

Considering the pivotal role of skeletal muscle in glucose utilisation, we further investigated the insulin signalling pathway in muscle tissue of the offspring. The results indicated disrupted insulin signalling in mHFD offspring. Serine phosphorylation of IRS1 (Ser636) was significantly reduced to 26.33% (*p <* 0.0001) in the skeletal muscle of mHFD offspring compared with mCD offspring (Figure 1F). Similarly, AKT (Ser473) phosphorylation reduced by 50.4% (*p <* 0.01) (Figure 1F). A similar downward trend was also observed in female offspring (Supplementary Figure 3F). These findings suggested that maternal prepregnancy obesity leads to systemic and muscular insulin resistance in the offspring, with similar effects observed in both sexes.

### 3.2 Maternal Prepregnancy Obesity Reduces Oxidative Muscle Fibre in Offspring

**FIGURE 2:**
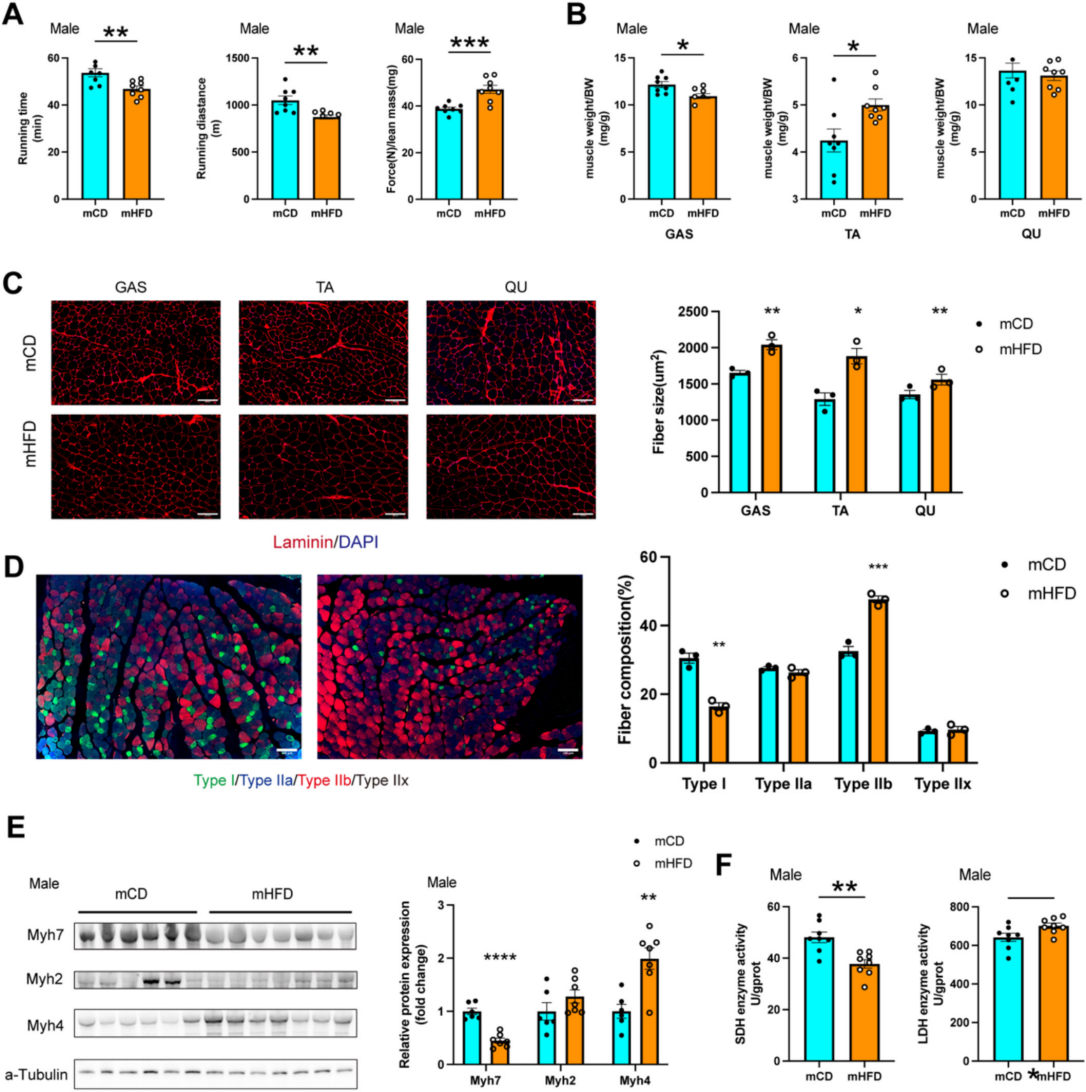
Maternal prepregnancy obesity alters muscle fibre composition in male offspring. (A–E) Assessment of muscle quality in male at 8 weeks of age. (A) Treadmill endurance performance after 3 days of treadmill exercise training, including running time, running distance and grip strength in mCD and mHFD mice (*n* = 8). (B) Relative weight of the gastrocnemius (GAS), tibialis anterior (TA) and quadriceps (QU) muscles (*n* = 6–7). (C) Representative laminin immunofluorescence images of GAS, TA and QU muscles (*n* = 3). At least 150 myofibers were analysed per experiment. Scale bar = 100 μm. (D) Representative immunofluorescence staining of fibre types (*n* = 3). Scale bar = 100 μm. (E) Western blot analysis and quantification of muscle fibre markers (*n* = 6–7). (F) SDH and LDH enzyme activity in male offspring from mCD and mHFD groups (*n* = 6). Unpaired Student's *t*‐test with two‐tailed distribution was used in data analyses. *p < 0.05, **p < 0.01, ***p < 0.001 and ****p < 0.0001.

To further investigate alterations in muscle tissue of offspring, we assessed the exercise capacity of both groups. The group of mHFD showed a greater muscle grip strength, shorter running duration and reduced time to exhaustion during running tests (Figure 2A and Supplementary Figure 5A). These findings indicated that mHFD performed better in explosive exercises but had diminished endurance during prolonged physical activity, suggesting a reduction of oxidative muscle fibres.

We observed that both male and female mice in the mHFD group had a higher tibialis anterior (TA) muscle weight, while no significant differences were found in gastrocnemius (GAS) and quadriceps (QU) muscle weights between the two groups (Supplementary Figure 4A,B). To better assess relative skeletal muscle mass changes, we calculated the ratio of muscle weight to body weight (Figure 2B and Supplementary Figure 5B). Further analysis revealed that the GAS ratio was significantly lower, whereas the TA ratio was significantly higher in both male and female mHFD mice. In contrast, the QU ratio showed no statistically significant difference between the groups.

Immunofluorescence staining showed that the average cross‐sectional area of individual muscle fibres was significantly larger in TA (1885 ± 183.9 μm^2^ vs. 1290 ± 149.3 μm^2^, *p <* 0.05), QU (1828 ± 110.4 μm^2^ vs. 1355 ± 99.75 μm^2^, *p <* 0.01), GAS (2043 ± 119.5 μm^2^ vs. 1655 ± 56.67 μm^2^, *p <* 0.01) in the mHFD group compared with the mCD group (Figure 2C and Supplementary Figure 5C). Furthermore, the staining for muscle fibre types showed that mHFD possessed a lower proportion of Type I fibres (8.66 ± 1.32% vs. 22.97 ± 1.31%, *p <* 0.001) and a higher percentage of Type IIb fibres (91.32 ± 1.32% vs. 77.37 ± 1.38%, *p <* 0.001) in the GAS in comparison with mCD (Figure 2D and Supplementary Figure 5D). In mHFD, the protein expression level of the slow‐twitch oxidative fibre marker Myh7 was significantly reduced. Concurrently, the expression of the fast‐twitch glycolytic fibre marker Myh4 was notably higher, while the expression of the fast‐twitch oxidative marker Myh2 remained unchanged (Figure 2E and Supplementary Figure 5E). As expected, the activity of the oxidative enzyme succinate dehydrogenase (SDH) was lower, while the activity of the glycolytic enzyme lactate dehydrogenase (LDH) was significantly higher in mHFD (Figure 2F and Supplementary Figure 5F). These results strongly suggested that maternal prepregnancy HFD exposure induced the transition of muscle fibre types, characterised by a reduction in slow‐twitch oxidative fibres and an increase in fast‐twitch glycolytic fibres.

### 3.3 Maternal Prepregnancy Obesity Alters IDH2 Expression in the Muscle Tissue of Offspring

**FIGURE 3:**
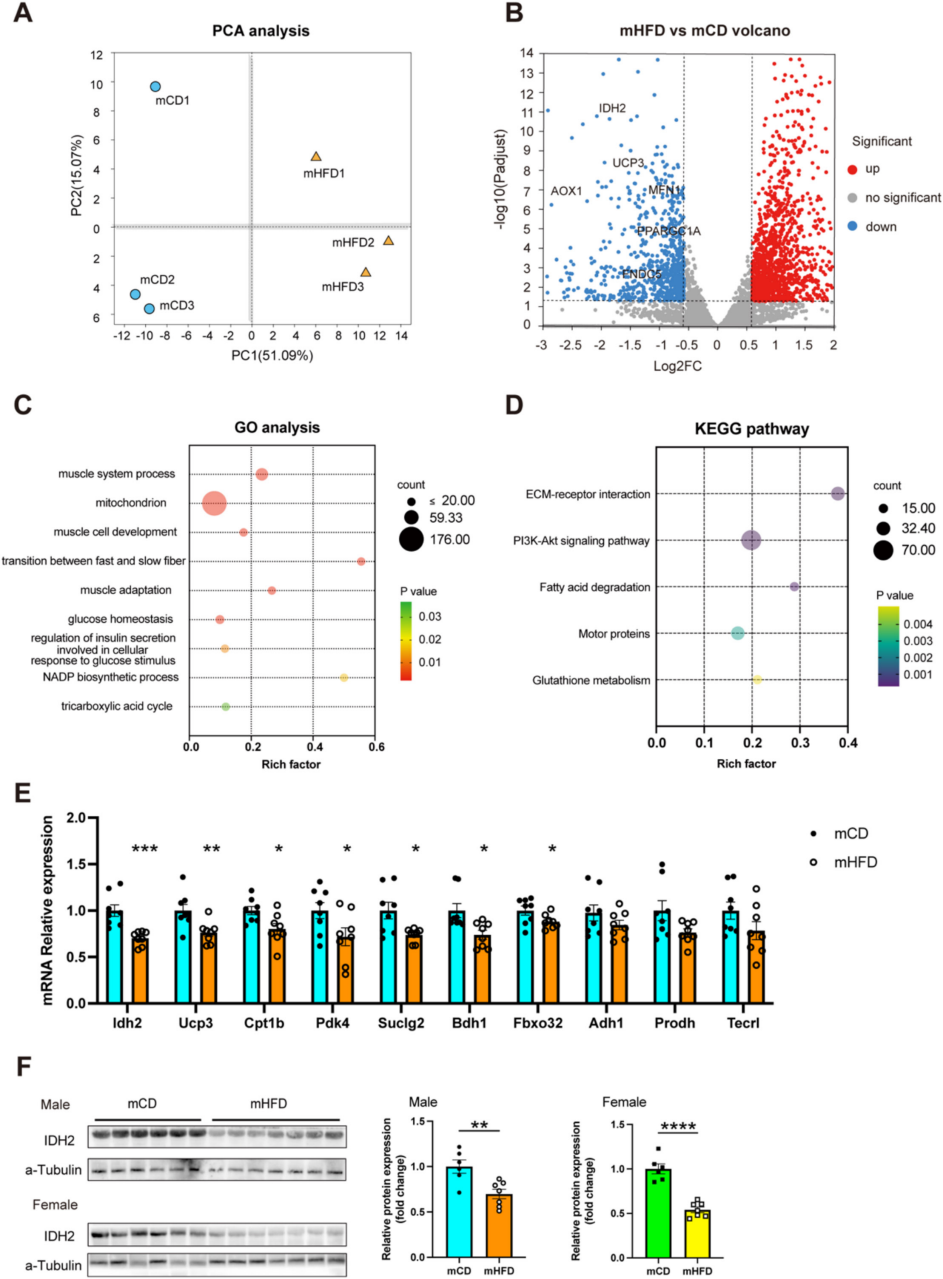
Maternal prepregnancy obesity alters the offspring expression of genes related to metabolism. (A) Principal component analysis (PCA) in mCD and mHFD. (B) Volcano plot showing the log2 fold‐change in mRNA expression in GAS of mCD and mHFD. Red and blue dots represent upregulated and downregulated differentially expressed genes (DEGs) a fold change with > 1.5 and adjusted *p* < 0.05, respectively. (C) Gene ontology analysis and Kyoto Encyclopedia of Genes and (D) Genomes (KEGG) pathway analysis of differentially expressed genes (DEGs) in muscle. (E) qPCR validation of 10 selected genes in muscle of offspring. (F) Protein expression level of IDH2 measured using WB analysis (*n* = 6–7). Unpaired Student's *t*‐test with two‐tailed distribution was used in data analyses. *p < 0.05, **p < 0.01, ***p < 0.001 and ****p < 0.0001.

To explore the underlying molecular mechanisms, we performed RNA sequencing (RNA‐seq) analysis on the GAS from both mCD and mHFD. Principal component analysis (PCA) revealed that PC1 and PC2 accounted for 51.09% and 15.07% of the total gene expression variance, respectively (Figure 3A). A total of 2170 differentially expressed genes were identified (Figure 3B). GO analysis showed that these genes were involved in multiple signalling pathways associated with biological processes and cellular component, such as muscle system process, mitochondrion, transition between fast and slow fibre and glucose homeostasis (Figure 3C). KEGG pathway analysis further indicated associations with pathways including ECM‐receptor interaction and PI3K‐Akt signalling pathway (Figure 3D). Because the skeletal muscle fibre characteristics of offspring from prepregnancy obese dams were primarily marked by a significant reduction in the proportion of slow‐twitch oxidative fibres, we focused our investigation on the screening and validation of downregulated genes. Because GO analysis revealed significant enrichment of mitochondrial function‐related gene sets, we ranked differentially expressed genes based on their log fold change and selected 10 highly altered candidate genes for validation using PCR. The selection criteria included genes that were both significantly downregulated (adjusted *p <* 0.05) and had the highest absolute log fold change values. Among these genes, mitochondrial NADP‐dependent isocitrate dehydrogenase 2 (IDH2) exhibited the most pronounced reduction in mRNA expression, showing a 29.67% lower expression in the mHFD group compared with the control mCD group. (Figure 3E). Additionally, we also observed significant differences in the protein levels of IDH2 between the two groups, with a 30.15% reduction in male offspring (*p <* 0.01) and a 46.02% reduction in female offspring (*p <* 0.0001), further supporting its potential role in the skeletal muscle of mHFD offspring (Figure 3F).

### 3.4 Muscle‐Specific IDH2 Regulates Systemic Insulin Resistance in mHFD by Modulating Muscle Fibre Type Transition

**FIGURE 4:**
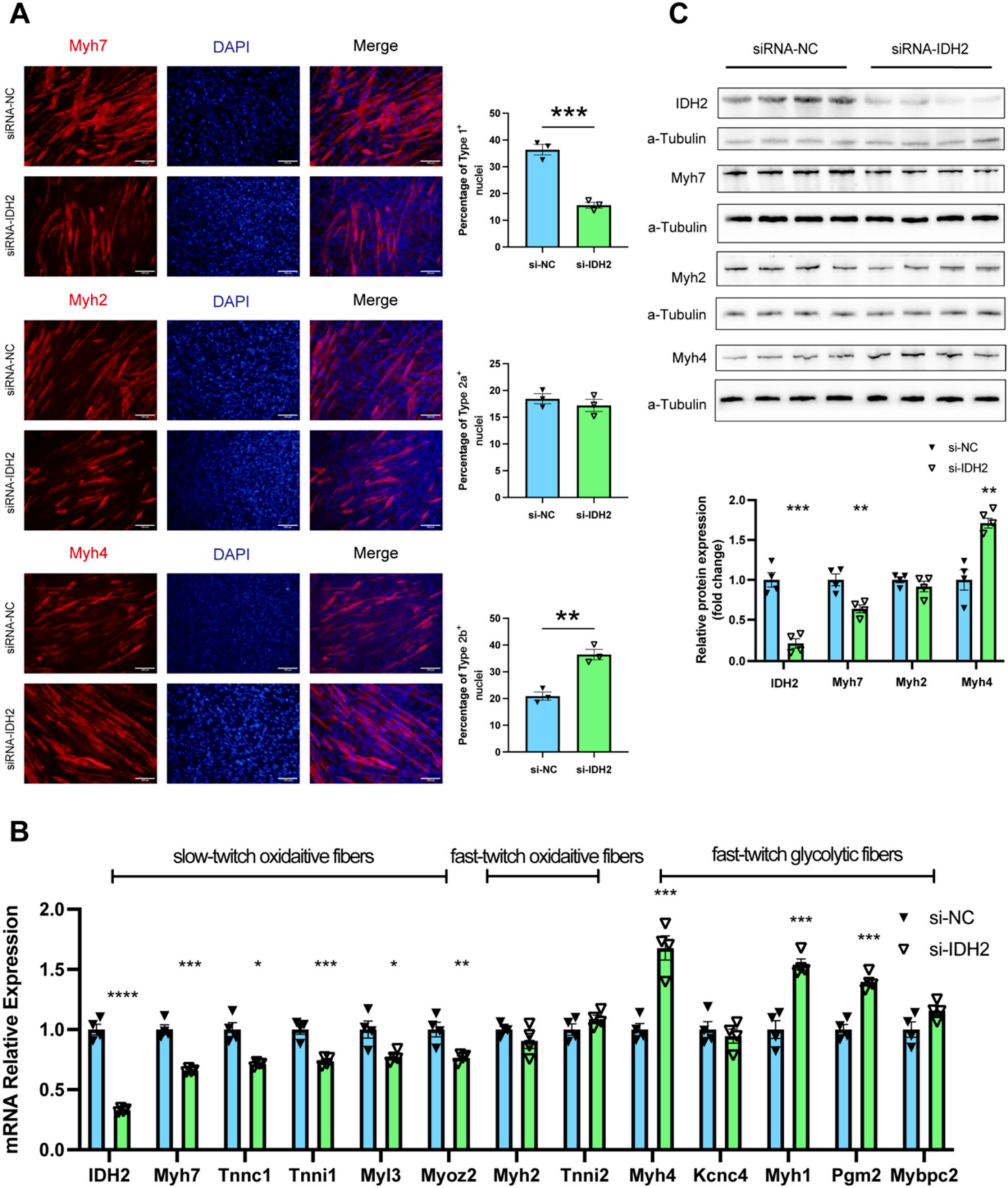
IDH2 regulates muscle fibre type transition in vitro. (A) Representative immunofluorescence images showing fibre type marker expression in C2C12 cells differentiated for 3 days. Scale bar = 200 μm. (B) Relative mRNA expression of markers of slow‐twitch oxidative fibres, fast‐twitch oxidative fibres and fast‐twitch glycolytic fibres was compared by qPCR (*n* = 4). (C) Protein levels of muscle fibre type markers in C2C12 cells differentiated for 3 days (*n* = 4). *p < 0.05, **p < 0.01, ***p < 0.001 and ****p < 0.0001.

To investigate the role of IDH2 in muscle fibre type transition, we performed an in vitro siRNA‐mediated knockdown of IDH2 (si‐IDH2) in the C2C12 skeletal muscle cell line. Remarkably, compared with cells transfected with the negative control (si‐NC), si‐IDH2 triggered the transition of muscle fibre types, with immunostaining evidence showing a significant reduction of the slow‐twitch oxidative fibres and a corresponding elevation of the fast‐twitch glycolytic fibres (Figure 4A). Further molecular studies revealed that the mRNA expression of Type I (oxidative slow‐twitch) fibre‐specific genes was significantly lower, including Myh7 (0.66‐fold, *p <* 0.001), Tnni1 (0.74‐fold, *p <* 0.001), Tnnc1(0.71‐fold, *p <* 0.05), Myl3 (0.77‐fold, *p <* 0.05) and Myoz2 (0.76‐fold, *p <* 0.01). The expression of Type IIa fibre‐specific genes, including Myh2 and Tnni2, showed no significant changes. In contrast, Type IIb fibre‐specific genes were significantly increased, including Myh4 (1.68‐fold, *p <* 0.001), Myh1 (1.54‐fold, *p <* 0.001) and Pgm2 (1.4‐fold, *p <* 0.001) (Figure 4B,C). These results underscored the critical regulatory role of IDH2 in muscle fibre type transition.

**FIGURE 5:**
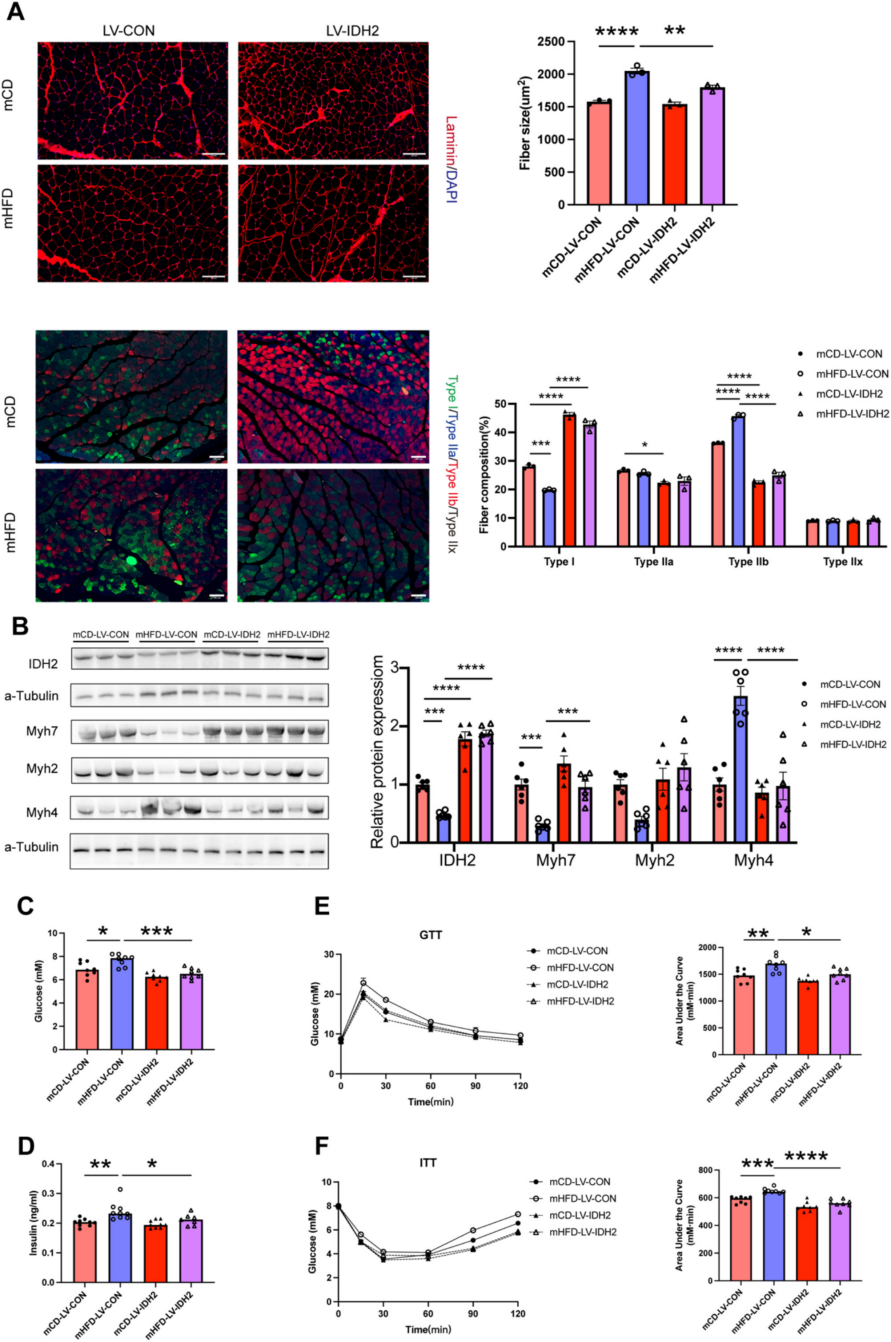
IDH2 regulates muscle fibre type transition and improves glucose metabolism in vivo. (A) Representative immunofluorescence images of laminin staining (upper panel) and fibre type staining (lower panel) in the GAS muscles from mCD and mHFD offspring injected with LV‐Control or LV‐IDH2 vectors. Scale bar = 200 μm. (B) Protein expression levels of muscle fibre markers (n = 6–7). Serum fasting glucose (C) and insulin levels (D) of mCD and mHFD offspring injected with LV‐Control or LV‐IDH2 vectors (*n* = 8–9). GTT (E) and ITT (F) of mCD and mHFD offspring injected with LV‐Control or LV‐IDH2 vectors (*n* = 8). Unpaired Student's *t*‐test with two‐tailed distribution was used in data analyses. *p < 0.05, **p < 0.01, ***p < 0.001 and ****p < 0.0001.

To further elucidate the role of IDH2 in muscle fibre transition and systemic metabolism in vivo, we utilised a tissue‐specific IDH2 overexpression approach using lentiviral vectors. Specifically, at the age of 4 weeks, either a control lentivirus (LV‐Control) or an IDH2 overexpressing lentivirus (LV‐IDH2) was injected into the GAS, TA and QU muscles of offspring. Four weeks after injection, the overexpression of IDH2 was observed to reduce the cross‐sectional area of muscle fibres in the GAS (Figure 5A). Notably, IDH2 overexpression partially restored the reduction of Type I fibres in mHFD, while simultaneously reducing the proportion of Type IIb fibres in the GAS (Figure 5A). Consistently, the overexpression of IDH2 significantly upregulated Myh7 expression (3.26‐fold, *p <* 0.001) and suppressed Myh4 expression (0.39‐fold, *p <* 0.0001) in mHFD (Figure 5B). More importantly, we found that muscle‐specific IDH2 overexpression reduced fasting glucose levels (7.675 ± 0.177 mmol/L vs. 6.55 ± 0.157 mmol/L, *p <* 0.01) and fasting insulin levels (0.24 ± 0.01 ng/mL vs. 0.21 ± 0.005 ng/mL, *p <* 0.05) in mHFD offspring (Figure 5C,D) and alleviated impaired glucose tolerance and insulin sensitivity (Figure 5E,F). These data significantly implied that muscular IDH2 ameliorates systemic insulin sensitivity in offspring exposed to maternal prepregnancy obesity, by facilitating the transition of muscle fibres from fast‐twitch glycolytic fibres to slow‐twitch oxidative fibres.

### 3.5 IDH2 Promotes Mitochondrial Biogenesis Through the Maintenance of Redox Homeostasis

Given the strong link between mitochondrial biogenesis and muscle fibre composition, we hypothesised that IDH2 modulates muscle fibre transition by maintaining redox homeostasis and enhancing PGC‐1α‐mediated mitochondrial biogenesis in offspring exposed to maternal prepregnancy obesity.

**FIGURE 6:**
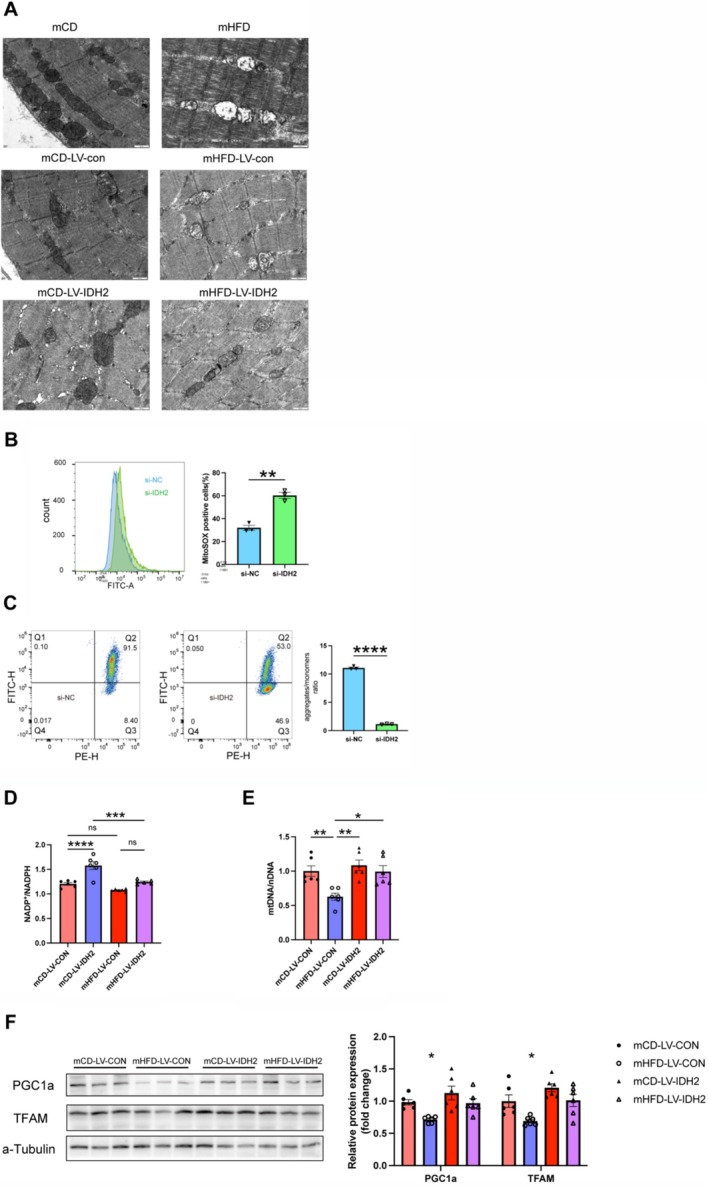
IDH2 influences muscle metabolism through mitochondrial biogenesis. (A) Representative transmission electron micrograph of GAS from mCD and mHFD injected with LV‐Control or LV‐IDH2 vectors (*n* = 3). (B) Mitochondrial reactive oxygen species (ROS) levels detected by flow cytometry (n = 3). (C) Mitochondrial membrane potential (MMP) measured by flow cytometry (*n* = 3). (D) NADP+/NADPH levels in skeletal muscle of mCD and mHFD injected with LV‐Control or LV‐IDH2 vectors (*n* = 6). (E) mtDNA content in the GAS muscles of mCD and mHFD injected with LV‐Control or LV‐IDH2 vectors (*n* = 6). (F) Protein expression levels of mitochondrial biogenesis‐related genes in GAS muscles of mCD and mHFD injected with LV‐Control or LV‐IDH2 vectors (*n* = 6). Unpaired Student's *t*‐test with two‐tailed distribution was used in data analyses. *p < 0.05, **p < 0.01, ***p < 0.001 and ****p < 0.0001.

To investigate mitochondrial abnormalities in mHFD offspring, we examined mitochondrial morphology, copy number and biogenesis‐related gene expression. Electron microscopic analysis of GAS muscle revealed that mHFD exhibited mitochondrial swelling, disrupted cristae structure and reduced matrix density (Figure 6A). Compared with mCD, mitochondrial DNA (mtDNA) copy number was significantly reduced in mHFD offspring (0.69‐fold, *p <* 0.01), suggesting impaired mitochondrial biogenesis (Supplementary Figure 6A). Furthermore, mRNA expression levels of key mitochondrial biogenesis markers were significantly lower in mHFD offspring, including PGC‐1α (0.71‐fold, *p <* 0.01), TFAM (0.79‐fold, *p <* 0.05), TFB1m (0.77‐fold, *p <* 0.05) and TFB2m (0.78‐fold, *p <* 0.05), confirming impaired mitochondrial replication and transcription (Supplementary Figure 6B).

To further investigate the mechanisms by which IDH2 impacts mitochondrial function, we transfected C2C12 cells with si‐IDH2. Flow cytometry revealed that compared with si‐NC, C2C12 cells treated with si‐IDH2 exhibited a significant increase in mitochondrial reactive oxygen species (ROS) levels (60.3 ± 2.65% in si‐IDH2 vs. 32.0 ± 2.265% in si‐NC, *p* < 0.01), coupled with a marked reduction in mitochondrial membrane potential, suggesting increased oxidative stress (Figure 6B,C). Consistently, we also observed that IDH2 knockdown led to an increase in the NADP^+^/NADPH ratio (1.62 ± 0.071 in si‐IDH2 vs. 1.22 ± 0.121 in si‐NC, *p* < 0.01) in cells (Supplementary Figure 6C). As expected, the expressions of the key mitochondrial biogenesis regulators, PGC1α and TFAM mRNA (Supplementary Figure 6D) and protein levels (Supplementary Figure 6E) were significantly decreased in si‐IDH2‐treated cells.

To confirm the role of IDH2 in mitochondrial biogenesis, we performed in vivo muscle‐specific lentiviral overexpression of IDH2 in mHFD. IDH2 overexpression improved mitochondrial morphology, as evidenced by more compact cristae structure and enriched mitochondrial matrix density (Figure 6A). NADP+/NADPH levels were restored following IDH2 overexpression in skeletal muscle, confirming the role of IDH2 in maintaining redox homeostasis (Figure 6D). Consistently, IDH2 overexpression in mHFD significantly increased mitochondrial DNA content (Figure 6E) and restored the expression levels of PGC1α and TFAM (Figure 6F). These results suggest that IDH2 may promote mitochondrial biogenesis by regulating redox homeostasis, ultimately affecting fibre type composition in skeletal muscle.

To elucidate the underlying cause of reduced IDH2 levels in mHFD, we further analysed the expression levels of IDH2 and genes associated with mitochondrial biogenesis in foetal muscle. Compared with mCD, the mRNA expression of IDH2 was significantly lower in muscle tissue of mHFD (Supplementary Figure 6F). Consistently, mtDNA content (0.67‐fold, *p <* 0.001) (Supplementary Figure 6G), as well as the expression levels of PGC1α (0.71‐fold, *p <* 0.01), TFAM (0.72‐fold, *p <* 0.05), TFB1m (0.75‐fold, *p <* 0.05) and TFB2m (0.77‐fold, *p <* 0.05) were reduced in the foetal muscle of mHFD (Supplementary Figure 6F). These results strongly implied an intra‐uterine epigenetic modulation of IDH2 in offspring exposed to maternal prepregnancy obesity.

### 3.6 Prepregnancy Histone 3 Lysine 9 Trimethylation Modification in Oocyte Contributes to IDH2 Gene Downregulation in Muscle Tissue of mHFD

**FIGURE 7:**
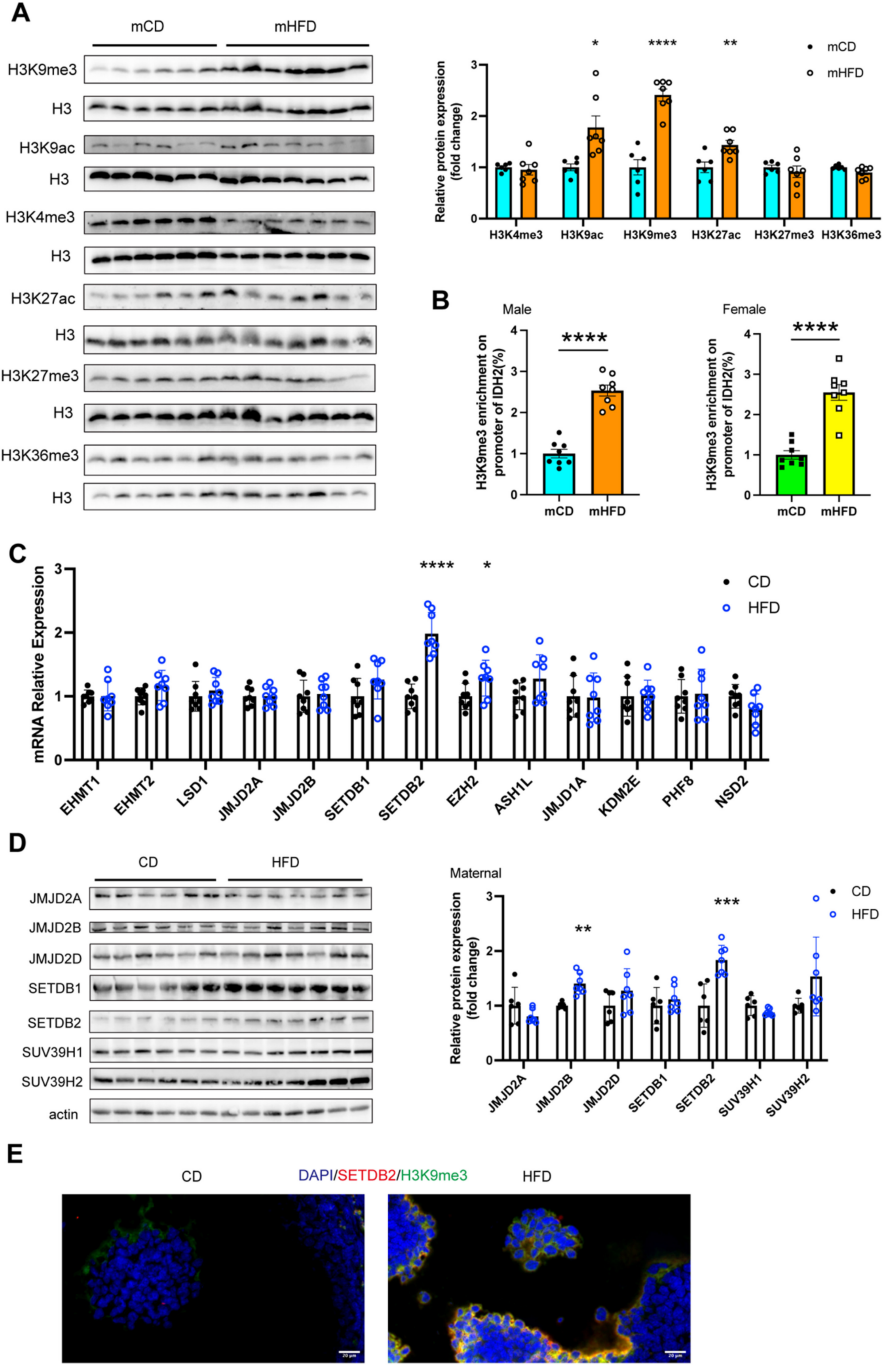
SETDB2‐mediated H3K9me3 modification suppresses IDH2 expression in mHFD offspring. (A) Representative Western blots images showing multiple histone modifications in skeletal muscle of offspring (*n* = 6–7). (B) H3K9me3 levels at the IDH2 promoters in skeletal muscle (*n* = 8). (C–D) mRNA and protein expression of histone methylation‐related gene expression in ovary of CD and HFD mothers (*n* = 8). (E) Representative immunofluorescent staining of SETDB2 and H3K9me3 in ovaries of CD and HFD mothers (*n* = 3). Scale bar = 20 μm. Unpaired Student's *t*‐test with two‐tailed distribution was used in data analyses. *p < 0.05, **p < 0.01, ***p < 0.001 and ****p < 0.0001.

Histone modifications, particularly acetylation and methylation, are crucial for intra‐uterine epigenetic control of protein expression. Acetylation of lysine 9 and lysine 27 on histone H3 (H3K9ac and H3K27ac) is associated with transcriptional activation, while tri‐methylation of histone H3 on lysine 9, lysine 27 and lysine 36 (H3K9me3, H3K27me3 and H3K36me3) leads to transcriptional repression [25]. We conducted analysis of histone modifications in the skeletal muscle of mCD and mHFD. Compared with mCD, the mHFD exhibited a pronounced elevation in the levels of H3K9ac (1.78‐fold, *p <* 0.05), H3K9me3 (2.41‐fold, *p <* 0.0001) and H3K27ac (1.43‐fold, *p <* 0.01), whereas the levels of H3K4me3, H3K36me3 and H3K36me3 remained unchanged (Figure 7A). Given that IDH2 expression was lower in mHFD mice, we focused on the investigation of H3K9me3, which is implicated in transcriptional silencing. ChIP‐qPCR analysis revealed that H3K9me3 enrichment at the IDH2 promoter was higher in both male (2.54‐fold, *p <* 0.0001) and female (2.55‐fold, *p <* 0.0001) mHFD mice (Figure 7B).

Given that maternal environmental exposures can alter epigenetic profiles of gametes and early embryos, we hypothesised that the origin of H3K9me3 modification might trace back to the gamete stage. We analysed the expression levels of several histone methyltransferases (EHMT1, EHMT2, JMJD2A, JMJD2B, SETDB1, SETDB2, EZH2, ASH1L, JMJD1A, NSD2) and demethylases (KDM5A and PHF8) in the ovaries of CD and HFD dams. Among these, the expression level of the histone methyltransferases SETDB2 was significantly higher in HFD dams (Figure 7C,D). Moreover, the increased expression of SETDB2 was co‐localised with the H3K9me3 histone modification in ovaries of HFD dams (Figure 7E). Collectively, our results suggested that reduced IDH2 expression in muscle tissue of mHFD may be attributed to H3K9me3 histone modification, which was likely initiated by the upregulated expression of SETDB2 in the oocytes of HFD dams.

## 4 Discussion

Maternal overnutrition increases the risk of metabolic syndrome in the offspring [3, 4, 5, 6, 7, 8, 9, 10, 11, 12]. The composition of muscle fibre types plays a critical role in both systemic and muscle‐specific metabolism [26]. However, the impact of prepregnancy obesity alters muscle fibre transition in offspring remains largely unexplored. Here, we observed that maternal prepregnancy HFD exposure led to a reduction in slow‐twitch oxidative muscle fibres in offspring without gender differentiation, accompanied with mitochondrial dysfunction in muscle tissue. Further investigation revealed that IDH2 was responsible for mitochondrial dysfunction, manipulating muscle fibre type transition. Restoring IDH2 expression in muscle tissue significantly enhanced the mitochondrial function, improved systemic and muscular insulin sensitivity and elevated the proportion of slow‐twitch oxidative muscle fibres. Moreover, we discovered significant enrichment of H3K9me3 at the IDH2 promoter region in the muscle tissue of offspring exposed to prepregnancy obesity, which could be originated from the increased H3K9me3 levels in the oocytes from HFD‐fed dams. Our findings highlight the intergenerational epigenetic mechanisms underlying the influence of prepregnancy obesity on muscle metabolism in offspring. To the best of our knowledge, this is the first study to report the impact of prepregnancy obesity on muscle fibre type transition muscle metabolism in offspring.

Offspring of mothers with prepregnancy obesity face an increased risk of obesity and type 2 diabetes (T2D), yet the underlying mechanisms remain elusive. Skeletal muscle insulin resistance, along with impaired glucose uptake, is a key factor driving the development of T2D in obesity, with alterations in muscle fibre composition playing a crucial role in this process [13, 14, 26, 27]. Slow‐twitch oxidative fibres are rich in mitochondria, providing superior oxidative capacity and utilisation of glucose and fatty acids. In contrast, fast‐twitch glycolytic fibres rely more on glycolytic metabolism with lower oxidative capacity [15, 16, 28]. Thus, muscles predominantly composed of slow‐twitch fibres exhibit greater insulin sensitivity. Patients with T2D or obesity exhibit a reduction in slow‐twitch oxidative fibres and a higher proportion of fast‐twitch glycolytic fibres [17, 18]. We found that, compared with mCD, the net weight of the TA was increased in the mHFD group, while the cross‐sectional area of individual muscle fibres was significantly larger in both male and female offspring of the mHFD group. Similarly, we observed fibre type transition (slow‐to‐fast shift) along with impaired systemic and muscular insulin sensitivity in offspring exposed to maternal prepregnancy obesity. Skeletal muscle is a highly heterogeneous and dynamic tissue, capable of adjusting fibre type composition and size in response to metabolic and functional demands [29]. Therefore, we propose that muscle fibre type transition in this model primarily represents an adaptive response to metabolic stress. Our findings highlight the reprogramming effects of prepregnancy obesity on muscle metabolism and muscle fibre type transitions in offspring.

RNA sequencing revealed reduced IDH2 expression. IDH2, a critical mitochondrial oxidase, catalyses the oxidative decarboxylation of isocitrate to α‐ketoglutarate, thereby generating NADPH [30]. It has been shown to play an essential role in regulating mitochondrial redox homeostasis and mitigating oxidative stress‐induced damage [30, 31]. The distinctively high mitochondrial density observed in oxidative muscle fibres underscored the potential significance of IDH2 in the regulation of muscle fibre type transition. Recent studies have identified an increasing number of mitochondria‐associated proteins as key regulators of myofiber composition, including peroxisome proliferator‐activated receptor‐γ coactivator 1α (PGC‐1α), AMP‐activated protein kinase (AMPK) and sirtuin 6 (Sirt6) [32, 33, 34, 35, 36]. Among these, PGC‐1α serves as a master regulator of mitochondrial biogenesis and plays a critical role in promoting the formation of oxidative muscle fibres [28, 37]. Furthermore, a decline in intracellular redox homeostasis and an increase in ROS reduce PGC1α protein stability [38]. In line with this, IDH2 knockdown led to a reduction in slow‐twitch oxidative fibres, accompanied by mitochondrial abnormalities, including disrupted morphology, redox homeostasis imbalance, impaired biogenesis and suppressed expression of key mitochondrial regulators PGC1α and TFAM—indicating that IDH2 deficiency disrupts mitochondrial biogenesis via oxidative stress‐mediated PGC‐1α suppression. More importantly, overexpression of IDH2 in mHFD not only restored the proportion of slow‐twitch oxidative fibres but also significantly improved their glucose tolerance and insulin sensitivity. These findings highlight the crucial role of the IDH2‐PGC1α signalling pathway in regulating skeletal muscle metabolism in the offspring of prepregnancy obese mothers.

Studies have shown that T2D is highly heritable [39, 40][S1]. However, genetic variants explain only a small portion of its heritability, emphasising the potential significance of epigenetics, particularly in the context of parental nutritional status correlates with offspring metabolic traits [3]. The harmful effects of maternal obesity may begin as early as the oocyte [S2, S3]. This early influence can affect both oocyte quality and its ability to support embryonic development [S4]. Notably, histone modifications are not completely erased during gametogenesis and fertilisation, particularly in regions of silent chromatin, where methylated histones can be retained through cell division [S5‐S7]. In this study, we detected enrichment of H3K9me3 at the IDH2 promoter in mHFD skeletal muscle, leading to a reduction in IDH2 expression. Consistently, we observed a significant increase in H3K9me3 levels in oocytes of obese dams, along with elevated expression of the histone methyltransferase SETDB2. These results suggest that increased H3K9me3 levels induced by SETDB2 in oocytes of obese dams may be transmitted to offspring, leading to reduced IDH2 expression, which further impairs mitochondrial function, reduces slow‐twitch oxidative muscle fibre composition and contributes to systemic insulin resistance. Our findings underscore the significance of transgenerational epigenetic inheritance of metabolic diseases through histone modifications.

This study has several limitations. First, we did not assess skeletal muscle function in F2 and F3 offspring, making it difficult to determine whether this epigenetic marker can be transmitted across multiple generations. Second, although we demonstrated the role of IDH2‐PGC1α signalling in prepregnancy obese skeletal muscle metabolism, other pathways may also be involved. Additionally, we did not analyse other metabolic organs, such as the liver, adipose tissue and pancreas, which may collectively influence offspring health.

In conclusion, our study reveals that increased H3K9me3 levels in oocytes of obese mothers could be intergenerationally transmitted to offspring, inhibiting the post‐transcriptional expression of IDH2, which further induces mitochondrial dysfunction, muscle fibre type transition and ultimately leads to impaired systemic insulin sensitivity. This work provides new perspectives on the development of metabolic diseases and identifies a potential therapeutic target for skeletal muscle insulin resistance, emphasising the critical importance of prepregnancy weight management for offspring health.

## Conflicts of Interest

The authors declare no conflicts of interest.

## Supporting information


**Figure S1** Maternal prepregnancy obesity model established after 8 weeks of high‐fat diet feeding. (A–F) Physiological and metabolic parameters measured before mating: (A) body mass, (B) serum cholesterol and serum triglycerides, (C) liver cholesterol and liver triglycerides, (D) serum glucose and (E) serum insulin (*n* = 8 per group). Data are presented as mean ± SEM. Unpaired Student’s *t*‐test with two‐tailed distribution was used in data analyses. *p < 0.05, **p < 0.01, ***p < 0.001 and ****p < 0.0001.
**Figure S2** Maternal prepregnancy obesity does not affect litter size or survival rate. (A) Litter size and (B) survival rate of offspring born from CD and HFD mothers (*n* = 8). Data are presented as mean ± SEM. Unpaired Student’s *t*‐test with two‐tailed distribution was used in data analyses. *p < 0.05, **p < 0.01, ***p < 0.001 and ****p < 0.0001.
**Figure S3.** Maternal prepregnancy obesity induces glucose intolerance and alters insulin signalling in female offspring. (A–G) Metabolic parameters were measured in mCD and mHFD female offspring (*n* = 8). (A) Body weight, (B) serum fasting glucose, (C) serum insulin. (D) glucose tolerance test (GTT) performed after an 8‐h fast and (E) insulin tolerance test (ITT) performed after a 6‐h fast. (F) Western blot analysis of tyrosine phosphorylation of IRS1(Ser636) and serinephosphorylation of AKT (Ser473) in female offspring muscle (*n* = 6–7). Data are presented as mean ± SEM. Unpaired Student’s *t*‐test with two‐tailed distribution was used in data analyses. *p < 0.05, **p < 0.01, ***p < 0.001 and ****p < 0.0001.
**Figure S4** Muscle wet weight in male and female offspring. (A) Muscle wet weight of gastrocnemius (GAS), tibialis anterior (TA) and quadriceps (QU) in male offspring. (B) Muscle wet weight of gastrocnemius (GAS), tibialis anterior (TA) and quadriceps (QU) in female offspring. Data are presented as mean ± SEM, *n* = 8 *p < 0.05, **p < 0.01, ***p < 0.001 and ****p < 0.0001.
**Figure S5** Maternal prepregnancy obesity alters muscle fibre composition in female offspring. (A–E) Assessment of muscle mass in female at 8 weeks of age. (A) Treadmill endurance performance after 3 days of treadmill exercise training: running time, running distance, grip strength in mCD and mHFD mice (*n* = 8). (B) Relative weight of the gastrocnemius (GAS), tibialis anterior (TA) and quadriceps (QU) muscles (*n* = 6–7). (C) Representative laminin immunofluorescence images of GAS, TA and QU muscles (*n* = 3). At least 150 myofibers were analysed per experiment. Scale bar = 100 μm. (D) Representative immunofluorescence staining of fibre types. Composition of each myofiber were quantified (*n* = 3). Bar = 100 μm. (E) Western blotting and quantification of muscle fibre markers (*n* = 6–7). (F) SDH enzyme activity and LDH enzyme activity in mCD and mHFD female offspring (*n* = 6). Unpaired Student’s *t*‐test with two‐tailed distribution was used in data analyses. *p < 0.05, **p < 0.01, ***p < 0.001 and ****p < 0.0001.
**Figure S6** IDH2 influences muscle metabolism through mitochondrial biogenesis. (A) Mitochondrial DNA (mtDNA) content in skeletal muscle of 8‐week‐old offspring (*n* = 8). (B) mRNA expression of mitochondrial biogenesis genes in muscle of 8‐week‐old offspring (*n* = 8). (C) NADP+/NADPH levels in C2C12 cells (*n* = 4). (D) mRNA expression of mitochondrial biogenesis genes in si‐IDH2 and si‐NC myotubes differentiated for 3 days (*n* = 4). (E) Western blotting analyses of mitochondrial biogenesis genes in si‐IDH2 and si‐NC myotubes differentiated for 3 days (*n* = 4). (F) mRNA expression of mitochondrial biogenesis genes in muscle of 8‐week‐old and E18.5 offspring (*n* = 8). (G) mtDNA content of E18.5 offspring (*n* = 8). Unpaired Student’s *t*‐test with two‐tailed distribution was used in data analyses. *p < 0.05, **p < 0.01, ***p < 0.001 and ****p < 0.0001.
**Table S1** Critical commercial assays.
**Table S2** Antibodies.
**Table S3** qPCR primer sequences.
**Table S4** siRNA oligonucleotides and CHIP‐qPCR primer sequences.
**Table S5** The coding sequence of IDH2 overexpression lentiviral vector.

## References

[1] A. A. Creanga , P. M. Catalano , and B. T. Bateman , “Obesity in Pregnancy,” New England Journal of Medicine 387 (2022): 248–259. 35857661 10.1056/NEJMra1801040

[2] L. Poston , R. Caleyachetty , S. Cnattingius , et al., “Preconceptional and Maternal Obesity: Epidemiology and Health Consequences,” Lancet Diabetes and Endocrinology 4 (2016): 1025–1036. 27743975 10.1016/S2213-8587(16)30217-0

[3] O. J. Rando and R. A. Simmons , “I'm Eating for Two: Parental Dietary Effects on Offspring Metabolism,” Cell 161 (2015): 93–105. 25815988 10.1016/j.cell.2015.02.021 PMC4465102

[4] D. J. Hoffman , T. L. Powell , E. S. Barrett , and D. B. Hardy , “Developmental Origins of Metabolic Diseases,” Physiological Reviews 101 (2021): 739–795. 33270534 10.1152/physrev.00002.2020 PMC8526339

[5] K. M. Godfrey , R. M. Reynolds , S. L. Prescott , et al., “Influence of Maternal Obesity on the Long‐Term Health of Offspring,” Lancet Diabetes and Endocrinology 5 (2017): 53–64. 27743978 10.1016/S2213-8587(16)30107-3 PMC5245733

[6] J. Si , A. Y. Meir , X. Hong , et al., “Maternal Pre‐Pregnancy BMI, Offspring Epigenome‐Wide DNA Methylation, and Childhood Obesity: Findings From the Boston Birth Cohort,” BMC Medicine 21 (2023): 317. 37612641 10.1186/s12916-023-03003-5 PMC10463574

[7] N. Malti , H. Merzouk , S. A. Merzouk , et al., “Oxidative Stress and Maternal Obesity: Feto‐Placental Unit Interaction,” Placenta 35 (2014): 411–416. 24698544 10.1016/j.placenta.2014.03.010

[8] M. Lecorguillé , M. Schipper , A. O'Donnell , et al., “Parental Lifestyle Patterns Around Pregnancy and Risk of Childhood Obesity in Four European Birth Cohort Studies,” Lancet Global Health 11, no. Suppl 1 (2023): S5. 36866482 10.1016/S2214-109X(23)00090-6

[9] S. P. Gilley , K. K. Harrall , C. Friedman , et al., “Association of Maternal BMI and Rapid Infant Weight Gain With Childhood Body Size and Composition,” Pediatrics 151 (2023): e2022059244. 37016999 10.1542/peds.2022-059244 PMC11033707

[10] L. Zhao , N. C. Law , N. A. Gomez , et al., “Obesity Impairs Embryonic Myogenesis by Enhancing BMP Signaling Within the Dermomyotome,” Advanced Science (Weinheim) 8 (2021): e2102157. 10.1002/advs.202102157 PMC8596142 34647690

[11] Y. T. Chen , Q. Y. Yang , Y. Hu , et al., “Imprinted lncRNA Dio3os Preprograms Intergenerational Brown Fat Development and Obesity Resistance,” Nature Communications 12 (2021): 6845. 10.1038/s41467-021-27171-1 PMC8617289 34824246

[12] B. Chen , Y. R. Du , H. Zhu , et al., “Maternal Inheritance of Glucose Intolerance Via Oocyte TET3 Insufficiency,” Nature 605 (2022): 761–766. 35585240 10.1038/s41586-022-04756-4

[13] T. Setiawan , I. N. Sari , Y. T. Wijaya , et al., “Cancer Cachexia: Molecular Mechanisms and Treatment Strategies,” Journal of Hematology & Oncology 16 (2023): 54. 37217930 10.1186/s13045-023-01454-0 PMC10204324

[14] R. A. DeFronzo and D. Tripathy , “Skeletal Muscle Insulin Resistance Is the Primary Defect in Type 2 Diabetes,” Diabetes Care 32, no. Suppl 2 (2009): S157–S163. 19875544 10.2337/dc09-S302 PMC2811436

[15] Y. J. Bahn , H. Yadav , P. Piaggi , et al., “CDK4‐E2F3 Signals Enhance Oxidative Skeletal Muscle Fiber Numbers and Function to Affect Myogenesis and Metabolism,” Journal of Clinical Investigation 133 (2023): e162479. 37395281 10.1172/JCI162479 PMC10313363

[16] W. Bai , Y. Zhang , J. Ma , et al., “FHL3 Promotes the Formation of Fast Glycolytic Muscle Fibers by Interacting With YY1 and Muscle Glycolytic Metabolism,” Cellular and Molecular Life Sciences 80 (2023): 27. 36602641 10.1007/s00018-022-04680-w PMC11073127

[17] Y. Duan , F. Li , B. Tan , K. Yao , and Y. Yin , “Metabolic Control of Myofibers: Promising Therapeutic Target for Obesity and Type 2 Diabetes,” Obesity Reviews 18 (2017): 647–659. 28391659 10.1111/obr.12530

[18] P. H. Albers , A. J. Pedersen , J. B. Birk , et al., “Human Muscle Fiber Type‐Specific Insulin Signaling: Impact of Obesity and Type 2 Diabetes,” Diabetes 64 (2015): 485–497. 25187364 10.2337/db14-0590

[19] C. E. McCurdy , S. Schenk , B. Hetrick , et al., “Maternal Obesity Reduces Oxidative Capacity in Fetal Skeletal Muscle of Japanese Macaques,” JCI Insight 1 (2016): e86612. 27734025 10.1172/jci.insight.86612 PMC5053156

[20] N. Cao , C. Lan , C. Chen , et al., “Prenatal Lipopolysaccharides Exposure Induces Transgenerational Inheritance of Hypertension,” Circulation 146 (2022): 1082–1095. 36004643 10.1161/CIRCULATIONAHA.122.059891 PMC9529859

[21] E. Heard and R. A. Martienssen , “Transgenerational Epigenetic Inheritance: Myths and Mechanisms,” Cell 157 (2014): 95–109. 24679529 10.1016/j.cell.2014.02.045 PMC4020004

[22] R. Barrès and J. R. Zierath , “The Role of Diet and Exercise in the Transgenerational Epigenetic Landscape of T2DM,” Nature Reviews. Endocrinology 12 (2016): 441–451. 10.1038/nrendo.2016.87 27312865

[23] J. Xu , C. Li , and X. Kang , “The Epigenetic Regulatory Effect of Histone Acetylation and Deacetylation on Skeletal Muscle Metabolism‐A Review,” Frontiers in Physiology 14 (2023): 1267456. 38148899 10.3389/fphys.2023.1267456 PMC10749939

[24] J. Li , S. Zhang , C. Li , et al., “Endurance Exercise‐Induced Histone Methylation Modification Involved in Skeletal Muscle Fiber Type Transition and Mitochondrial Biogenesis,” Scientific Reports 14 (2024): 21154. 39256490 10.1038/s41598-024-72088-6 PMC11387812

[25] J. Park , K. Lee , K. Kim , and S. J. Yi , “The Role of Histone Modifications: From Neurodevelopment to Neurodiseases,” Signal Transduction and Targeted Therapy 7 (2022): 217. 35794091 10.1038/s41392-022-01078-9 PMC9259618

[26] N. Motohashi , A. Uezumi , A. Asakura , et al., “Tbx1 Regulates Inherited Metabolic and Myogenic Abilities of Progenitor Cells Derived From Slow‐ and Fast‐Type Muscle,” Cell Death and Differentiation 26 (2019): 1024–1036. 30154444 10.1038/s41418-018-0186-4 PMC6748120

[27] W. Campodonico‐Burnett , B. Hetrick , S. R. Wesolowski , et al., “Maternal Obesity and Western‐Style Diet Impair Fetal and Juvenile Offspring Skeletal Muscle Insulin‐Stimulated Glucose Transport in Nonhuman Primates,” Diabetes 69 (2020): 1389–1400. 32354857 10.2337/db19-1218 PMC7306120

[28] J. S. Son , S. A. Chae , H. Wang , et al., “Maternal Inactivity Programs Skeletal Muscle Dysfunction in Offspring Mice by Attenuating Apelin Signaling and Mitochondrial Biogenesis,” Cell Reports 33 (2020): 108461. 33264618 10.1016/j.celrep.2020.108461 PMC8137280

[29] A. S. Pereyra , C. T. Lin , D. M. Sanchez , et al., “Skeletal Muscle Undergoes Fiber Type Metabolic Switch Without Myosin Heavy Chain Switch in Response to Defective Fatty Acid Oxidation,” Molecular Metabolism 59 (2022): 101456. 35150906 10.1016/j.molmet.2022.101456 PMC8898976

[30] H. Wang , Q. Xiong , G. He , et al., “Hepatic IDH2 Regulates Glycolysis and Gluconeogenesis,” Metabolism 143 (2023): 155559. 37044373 10.1016/j.metabol.2023.155559

[31] J. H. Lee , Y. Go , D. Y. Kim , et al., “Isocitrate Dehydrogenase 2 Protects Mice From High‐Fat Diet‐Induced Metabolic Stress by Limiting Oxidative Damage to the Mitochondria From Brown Adipose Tissue,” Experimental & Molecular Medicine 52 (2020): 238–252. 32015410 10.1038/s12276-020-0379-z PMC7062825

[32] J. Li , Z. Zhang , H. Bo , and Y. Zhang , “Exercise Couples Mitochondrial Function With Skeletal Muscle Fiber Type via ROS‐Mediated Epigenetic Modification,” Free Radical Biology & Medicine 213 (2024): 409–425. 38295887 10.1016/j.freeradbiomed.2024.01.036

[33] J. Liu , X. Liang , D. Zhou , et al., “Coupling of Mitochondrial Function and Skeletal Muscle Fiber Type by a miR‐499/Fnip1/AMPK Circuit,” EMBO Molecular Medicine 8 (2016): 1212–1228. 27506764 10.15252/emmm.201606372 PMC5048369

[34] J. Lin , H. Wu , P. T. Tarr , et al., “Transcriptional Co‐Activator PGC‐1 Alpha Drives the Formation of Slow‐Twitch Muscle Fibres,” Nature 418 (2002): 797–801. 12181572 10.1038/nature00904

[35] H. Liang and W. F. Ward , “PGC‐1alpha: A key Regulator of Energy Metabolism,” Advances in Physiology Education 30 (2006): 145–151. 17108241 10.1152/advan.00052.2006

[36] T. Yasuda , T. Ishihara , A. Ichimura , and N. Ishihara , “Mitochondrial Dynamics Define Muscle Fiber Type by Modulating Cellular Metabolic Pathways,” Cell Reports 42 (2023): 112434. 37097817 10.1016/j.celrep.2023.112434

[37] M. Montori‐Grau , D. Aguilar‐Recarte , M. Zarei , J. Pizarro‐Delgado , X. Palomer , and M. Vázquez‐Carrera , “Endoplasmic Reticulum Stress Downregulates PGC‐1α in Skeletal Muscle Through ATF4 and an mTOR‐Mediated Reduction of CRTC2,” Cell Communication and Signaling: CCS 20 (2022): 53. 35428325 10.1186/s12964-022-00865-9 PMC9012021

[38] O. Abu Shelbayeh , T. Arroum , S. Morris , and K. B. Busch , “PGC‐1α Is a Master Regulator of Mitochondrial Lifecycle and ROS Stress Response,” Antioxidants (Basel) 12 (2023): 1075. 37237941 10.3390/antiox12051075 PMC10215733

[39] C. Fuchsberger , J. Flannick , and T. M. Teslovich , “The Genetic Architecture of Type 2 Diabetes,” Nature 536 (2016): 41–47. 27398621 10.1038/nature18642 PMC5034897

[40] C. Davegårdh , S. García‐Calzón , K. Bacos , and C. Ling , “DNA Methylation in the Pathogenesis of Type 2 Diabetes in Humans,” Molecular Metabolism 14 (2018): 12–25. 29496428 10.1016/j.molmet.2018.01.022 PMC6034041

