# Supplementary material for: Prepregnancy Obesity Reprograms Offspring Skeletal Muscle Fibre Transition Through H3K9me3
Source: J Cachexia Sarcopenia Muscle. 2025 Apr 21;16(2):e13825. doi: 10.1002/jcsm.13825 (PMC12010212; doi:10.1002/jcsm.13825)
Supplement: Supplementary file 1 — Figure S1 Maternal prepregnancy obesity model established after 8 weeks of high‐fat diet feeding. (A–F) Physiological and metabolic parameters measured before mating: (A) body mass, (B) serum cholesterol and serum triglycerides, (C) liver cholesterol and liver triglycerides, (D) serum glucose and (E) serum insulin (n = 8 per group). Data are presented as mean ± SEM. Unpaired Student’s t‐test with two‐tailed distribution was used in data analyses. *p < 0.05, **p < 0.01, ***p < 0.001 and ****p < 0.0001. Figure S2 Maternal prepregnancy obesity does not affect litter size or survival rate. (A) Litter size and (B) survival rate of offspring born from CD and HFD mothers (n = 8). Data are presented as mean ± SEM. Unpaired Student’s t‐test with two‐tailed distribution was used in data analyses. *p < 0.05, **p < 0.01, ***p < 0.001 and ****p < 0.0001. Figure S3. Maternal prepregnancy obesity induces glucose intolerance and alters insulin signalling in female offspring. (A–G) Metabolic parameters were measured in mCD and mHFD female offspring (n = 8). (A) Body weight, (B) serum fasting glucose, (C) serum insulin. (D) glucose tolerance test (GTT) performed after an 8‐h fast and (E) insulin tolerance test (ITT) performed after a 6‐h fast. (F) Western blot analysis of tyrosine phosphorylation of IRS1(Ser636) and serinephosphorylation of AKT (Ser473) in female offspring muscle (n = 6–7). Data are presented as mean ± SEM. Unpaired Student’s t‐test with two‐tailed distribution was used in data analyses. *p < 0.05, **p < 0.01, ***p < 0.001 and ****p < 0.0001. Figure S4 Muscle wet weight in male and female offspring. (A) Muscle wet weight of gastrocnemius (GAS), tibialis anterior (TA) and quadriceps (QU) in male offspring. (B) Muscle wet weight of gastrocnemius (GAS), tibialis anterior (TA) and quadriceps (QU) in female offspring. Data are presented as mean ± SEM, n = 8 *p < 0.05, **p < 0.01, ***p < 0.001 and ****p < 0.0001. Figure S5 Maternal prepregnancy obesity alters muscle fibre [file JCSM-16-e13825-s001.docx]

**Supporting information**

**Pre-pregnancy Obesity Reprograms Offspring Skeletal Muscle Metabolism through H3K9me3**

Yichi Wu^1,2^, Sujuan Li^1,2^, Jingyi Zhang^1,2^, Anran Tian^1,2^, Xiangyao Wang^3^, Xi Yang^1,2^, Fucheng Meng^1,2^, Qing Li^1,2^, Yuan Gao^1,2^, Yingying Li^1,2^, Furong Liang^1,2^, Minglan Yao^1,2^, Xiaoping Luo^1,2^ and Cai Zhang^1,2^

1. Department of Pediatrics, Tongji Hospital, Tongji Medical College, Huazhong University of Science and Technology, Wuhan 430030, China

2. Hubei Key Laboratory of Pediatric Genetic Metabolic and Endocrine Rare Diseases, Wuhan 430030, China

3. Department of Stomatology, Tongji Hospital, Tongji Medical College, Huazhong University of Science and Technology, Wuhan 430030, China

**This document contains the following informaton:**

- **Supplementary Methods**
- **Supplementary Figures 1-6**
- **Supplementary Tables 1-5**
- **Supplementary References**

**Supplementary Methods**

**Immunofluorescence staining**

Muscle and ovary tissues were rapidly frozen in liquid nitrogen, fixed in 4% paraformaldehyde, dehydrated by gradient ethanol, embedded in paraffin, and sectioned at 6 μm. The tissue sections and C2C12 cell slides were blocked and permeabilized in blocking buffer containing 5% of BSA and 0.4% of Triton X-100 in PBS for 1 hour. After being blocked, the slides were incubated with primary antibodies (Table S2) overnight at 4°C. After being incubated with appropriate secondary antibodies at room temperature for 2 h, the sections were visualized and photographed using a fluorescence microscope (IX71, OLYMPUS, Japan). DAPI (Servicebio, China) was used for nuclear staining. The fiber cross-sectional area (CSA) and fiber type distribution were quantified by analyzing the thresholded images using ImageJ. The samples were transferred and prepared in ImageJ by converting the images to 8-bit, applying thresholding, and performing particle analysis to measure fiber size and distribution.

**Intraperitoneal glucose and insulin tolerance tests**

Mice were fasted for 8 hours for glucose tolerance test (GTT) and 6 hours for insulin tolerance test (ITT), during which time they were provided with water. Mice were intraperitoneally injected with 2 g/kg body weight of glucose or 0.75 U/kg body weight of insulin. Blood glucose was measured at 0, 15, 30, 60, 90, and 120 min after injection respectively.

**Western blot**

The samples (30 μg) were separated by SDS-PAGE and transferred to PVDF membranes (Millionpore, USA). After blocking with 5% skim milk, blots were incubated with the primary antibodies (Table S2) overnight at 4°C. Then, HRP-conjugated secondary antibodies were incubated for 2 h at room temperature. β-Tubulin /Histone 3 was used for normalization, and the intensity of each reactive band was analyzed using Image J software.

**RNA extraction and quantitative real-time PCR (qPCR)**

Total RNA was prepared from tissues using RNAiso Plus (Takara) following the manufacturer’s protocol. cDNA was synthesized using PrimeScript RT Master Mix Kit (Takara, Japan). Quantitative Real-time PCR was performed using CFX Connect Real-Time System (Bio-Rad, USA). The expression levels of each transcript were normalized to 18S rRNA for muscle samples and actin for ovarian samples. All primers were were listed in Supplementary Table 3.

**Mitochondrial DNA (mtDNA) copy number**

The muscle was digested overnight with Proteinase K, and DNA was extracted with Dneasy Tissue Kit (Qiagen, 69504). Mitochondria and nDNA were amplified by qPCR using 12S rRNA and 5S rRNA primers (Supplemental Table 3), respectively. mtDNA copy numbers were calculated by normalizing 12S rRNA to 5S rRNA levels.

**Cell culture, differentiation and transient transfection**

When C2C12 cells (CCTCC, China) and primary skeletal muscle cells density reached 80% - 90%, medium containing 2% horse serum (Solarbio, S9050, China) DMEM was used to induce myogenic differentiation and myotube fusion. For knockdown experiments, cells were transfected with siRNA using RNAimax (Thermofisher Scientific, USA) according to the manufacturer's instructions and the sequences used are shown in the Supplementary Table 4.

**RNA sequencing and data analysis**

RNA-seq was carried out by Majorbio (Shanghai, China). Total RNA was prepared from mCD and mHFD using TRIzol. Differentially expressed mRNAs were predicted also using DESeq2. Volcano plot of differentially expressed genes (DEGs) were created with adjusted p value <0.05. Genomes pathway, Gene Ontology (GO) enrichment and Kyoto Encyclopedia of Genes and Genomes (KEGG) analyses were performed using DAVID 6.8 on DEGs with adjusted p value <0.05.

**Transmission electron microscopy (TEM)**

Skeletal muscle tissues were cut into small pieces (1 mm³) and fixed in 3% glutaraldehyde at 4°C. The tissue blocks were then sectioned using a Leica ultramicrotome, and ultrathin sections (60 nm) were prepared. The sections were visualized by the presence of silver color shadowing. The sections were placed on copper grids and double-stained with uranyl acetate and lead citrate. The sections were observed using an H-7650 transmission electron microscope (Hitachi High Technologies).

**Flow cytometry**

The mitochondrial membrane potential (MN) of cells was determined by JC-1 (Beyotime, China) according to the instructions. Mitochondrial reactive oxygen species (mtROS) production of cells was measured by staining with MitoSOX™ Red (Thermofisher Scientific, USA). Briefly, C2C12 cells were rinsed twice with PBS and incubated with either 5 μg/mL JC-1 for 30 min or 2 μmol/L MitoSox for 30 min, then cells were washed twice with PBS and detected by flow cytometry (Beckman Coulter, USA). Flow cytometry data were analyzed by FlowJo software (Treestar Inc., San Carlos, CA). Gates were determined based on fluorescence minus control.

**Lentiviruses-mediated overexpression in muscles**

The coding sequence (CDS) of the mouse IDH2 was cloned into the pCDH-CMV-MCS-EF1a-puro-3flag lentiviral vector. mCD and mHFD male mice used for lentiviral injections from different litters were randomly selected. Lentiviral injections were administered to 4-week-old mCD and mHFD for 4 weeks, with weekly injections of negative control (LV-CON) or shRNA-mediated overexpression lentiviruses (LV-IDH2) in the tibialis anterior, gastrocnemius, and quadriceps muscles of the both legs, and the volume of the lentivirus for continuous injection is 50 μL, 100 μL, and 100 μL, the lentivirus concentration used in all assays was above 1 × 10^8^ transducing units per milliliter. The sequences used are shown in the Supplementary Table 5.

**Chromatin immunoprecipitation (ChIP)**

Skeletal muscle tissue chromatin precipitation was performed using Chromatin IP kit (Cell Signaling Technologies, 56383) following manufacturer’s instructions. In brief, 20 mg of frozen tissue was cross-linked in 1% formaldehyde in PBS for 10 minutes. Chromatin was sheared using Bioruptor UCD-200 (Diagenode Inc.) for 15 minutes (5 minutes per run with 30 seconds on 30 seconds off, in 2-minute intervals). Chromatin 10 to 20 μg was incubated with indicated antibodies overnight. The protein-DNA was then de–cross-linked. DNA was purified and subjected to qPCR. The sequences of IDH2 used for CHIP-qPCR are shown in the Supplementary Table 4.

**Supplementary Figures**
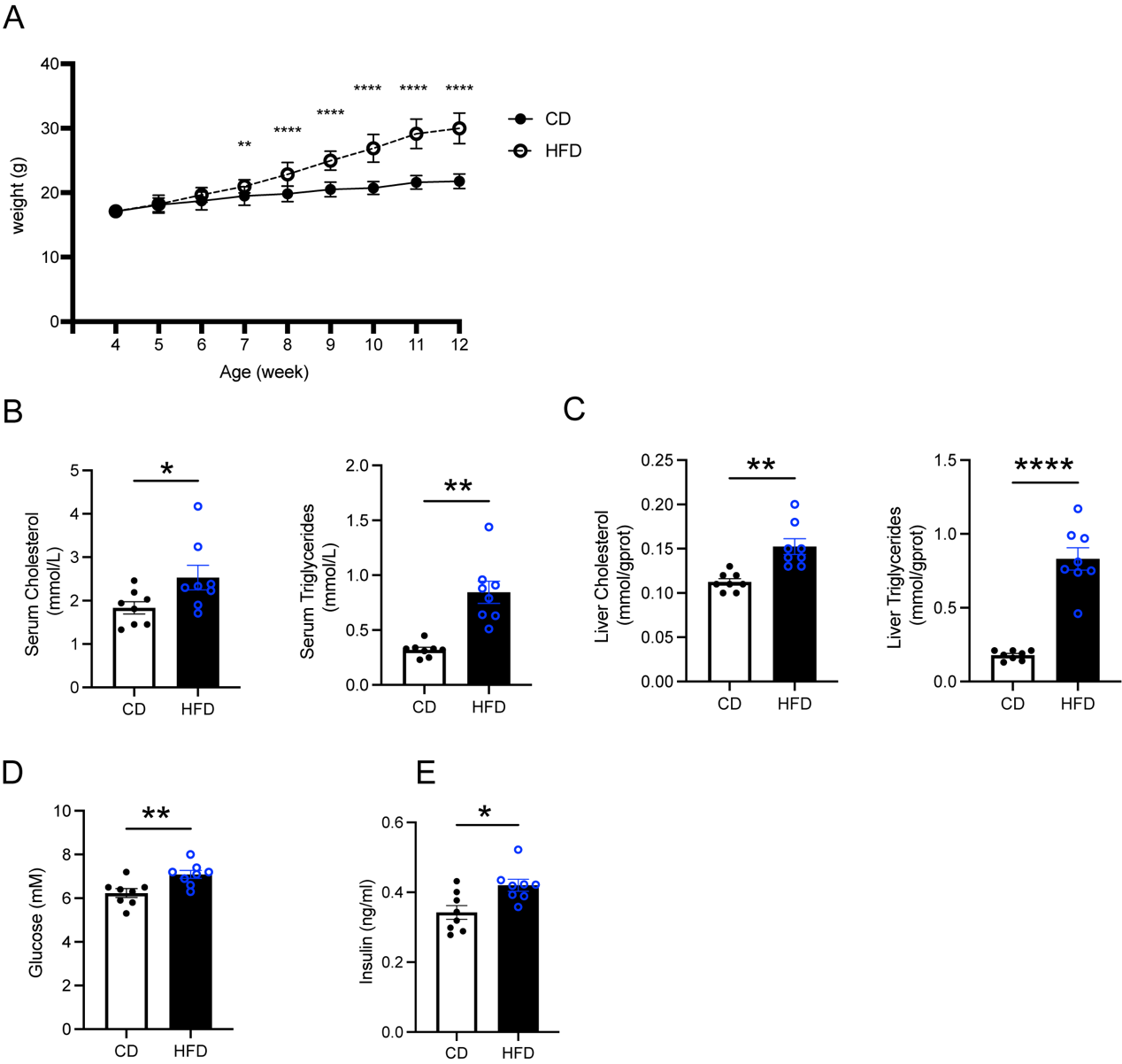


**Figure S1- Maternal pre-pregnancy obesity model established after 8-weeks of high-fat diet feeding.** (A-F) Physiological and metabolic parameters measured before mating: (A) Body mass, (B) Serum cholesterol and serum triglycerides, (C) Liver cholesterol and liver triglycerides, (D) Serum glucose, and (E) Serum insulin (n = 8 per group). Data are presented as mean ± SEM. Unpaired Student’s t-test with two-tailed distribution was used in data analyses. **P* < 0.05, ***P* < 0.01, ****P* < 0.001 and *****P* < 0.0001.


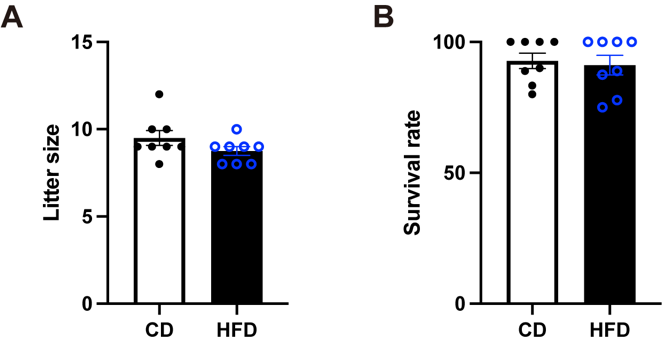


**Figure S2- Maternal pre-pregnancy obesity does not affect litter size or survival rate.** (A) Litter size and (B) survival rate of offspring born from CD and HFD mothers (n = 8). Data are presented as mean ± SEM. Unpaired Student’s t-test with two-tailed distribution was used in data analyses. **P* < 0.05, ***P* < 0.01, ****P* < 0.001 and *****P* < 0.0001.


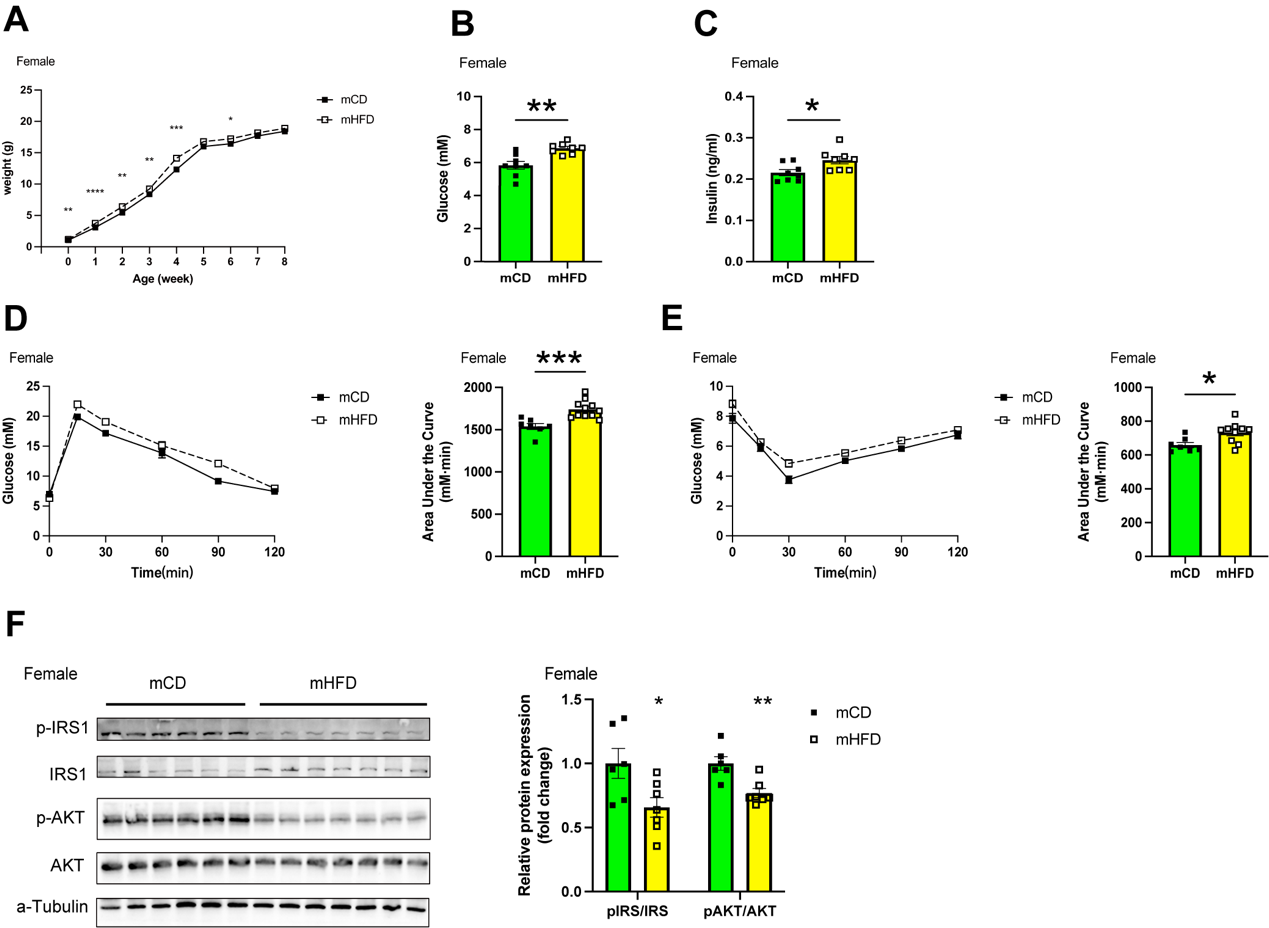


**Figure S3- Maternal pre-pregnancy obesity induces glucose intolerance and alters insulin signaling in female offspring**. (A-G) Metabolic parameters were measured in mCD and mHFD female offspring (n = 8). (A) Body weight, (B) serum fasting glucose, (C) serum insulin. (D) Glucose tolerance test (GTT) performed after an 8-hour fast, and (E) insulin tolerance test (ITT) performed after a 6-hour fast. (F) Western blot analysis of tyrosine phosphorylation of IRS1(Ser636) and serinephosphorylation of AKT (Ser473) in female offspring muscle (n = 6-7). Data are presented as mean ± SEM. Unpaired Student’s t-test with two-tailed distribution was used in data analyses. **P* < 0.05, ***P* < 0.01, ****P* < 0.001 and *****P* < 0.0001.


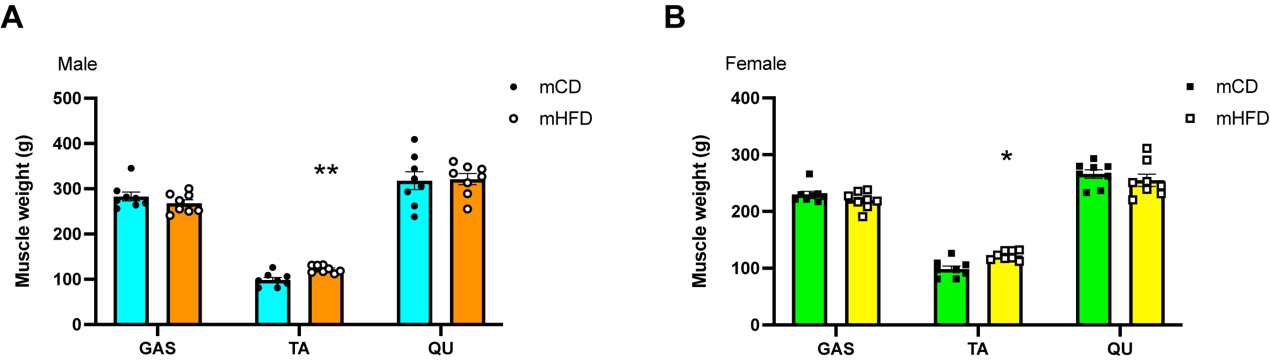


**Figure S4- Muscle wet weight in male and female offspring.**

(A) Muscle wet weight of gastrocnemius (GAS), tibialis anterior (TA), and quadriceps (QU) in male offspring. (B) Muscle wet weight of gastrocnemius (GAS), tibialis anterior (TA), and quadriceps (QU) in female offspring. Data are presented as mean ± SEM, n =8 **P* < 0.05, ***P* < 0.01, ****P* < 0.001 and *****P* < 0.0001.


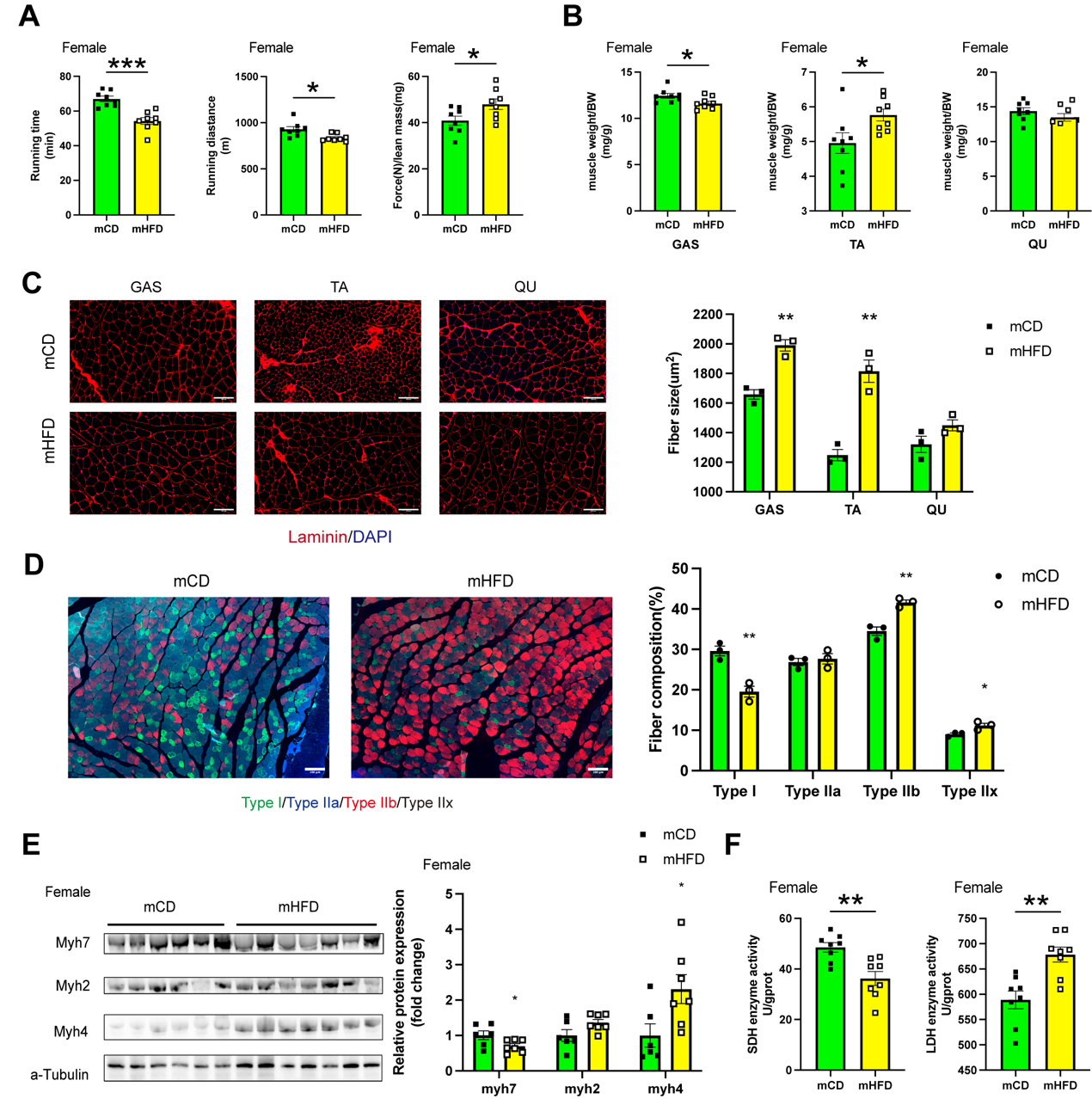


**Figure S5- Maternal pre-pregnancy obesity alters muscle fiber composition in female offspring.** A-E. Assessment of muscle mass in female at 8 weeks of age. (A) Treadmill endurance performance after 3 day of treadmill exercise training: running time, running distance, grip strength in mCD and mHFD mice (n = 8).  (B) Relative weight of the gastrocnemius (GAS), tibialis anterior (TA), and quadriceps (QU) muscles (n = 6-7).  (C) Representative laminin immunofluorescence images of GAS, TA, and QU muscles (n = 3). At least 150 myofibers were analyzed per experiment. Scale bar = 100 μm. (D) Representative immunofluorescence staining of fiber types. Composition of each myofiber were quantified (n = 3). Bar=100 μm. (E) Western blotting and quantification of muscle fiber markers (n = 6-7). (F) SDH enzyme activity and LDH enzyme activity in mCD and mHFD female offspring (n = 6). Unpaired Student’s t-test with two-tailed distribution was used in data analyses. **P* < 0.05, ***P* < 0.01, ****P* < 0.001 and *****P* < 0.0001.


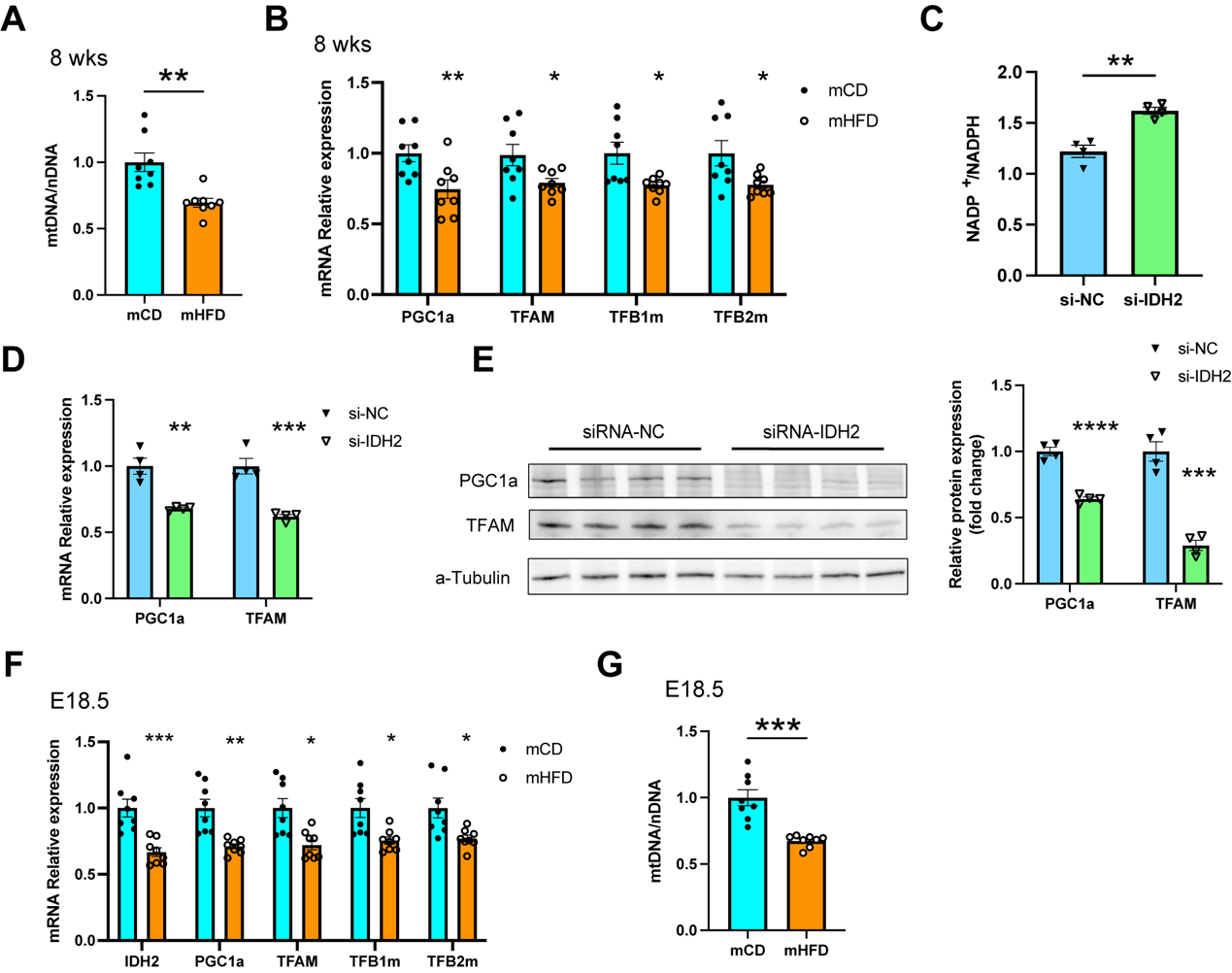


**Figure S6- IDH2 influences muscle metabolism through mitochondrial biogenesis.** (A) Mitochondrial DNA (mtDNA) content in skeletal muscle of 8-week-old offspring (n = 8). (B) mRNA expression of mitochondrial biogenesis genes in muscle of 8-week-old offspring (n = 8). (C) NADP+/NADPH levels in C2C12 cells (n = 4). (D) mRNA expression of mitochondrial biogenesis genes in si-IDH2 and si-NC myotubes differentiated for 3 days (n = 4). (E) Western blotting analyses of mitochondrial biogenesis genes in si-IDH2 and si-NC myotubes differentiated for 3 days (n = 4). **(**F) mRNA expression of mitochondrial biogenesis genes in muscle of 8-week-old and E18.5 offspring (n = 8). **(G)** mtDNA conten of E18.5 offspring (n = 8). Unpaired Student’s t-test with two-tailed distribution was used in data analyses. **P* < 0.05, ***P* < 0.01, ****P* < 0.001 and *****P* < 0.0001.

**Supplementary Table 1 Critical Commercial Assays**

| Reagent | Manufacturer | Catalog Number |
| --- | --- | --- |
| Triglyceride assay kit | Nanjing Jiancheng | A110-2-1 |
| Cholesterol assay kit | Nanjing Jiancheng | A111-2-1 |
| Insulin ELISA kit | Alpco | 80-INSMSU-E01 |
| Succinate Dehydrogenase assay kit | Elabscience | E-BC-K649-M |
| Lactate Dehydrogenase assay kit | Elabscience | E-BC-K046-M |
| NADP+/NADPH Colorimetric Assay Kit | Elabscience | E-BC-K803-M |

**Supplementary Table 2 Antibodies**

| Target | Manufacturer | Catalog Number | Dilution | Host Species |
| --- | --- | --- | --- | --- |
| IRS1 | CST | 2382S | 1:1000 | Rabbit |
| Phospho-IRS1 (Ser636) | Invitrogen | PA5-104876 | 1:1000 | Rabbit |
| AKT | Abcam | 192623 | 1:1000 | Rabbit |
| Phospho-AKT (Ser473) | CST | 4060S | 1:1000 | Rabbit |
| a-Tubulin | Abclonal | AC012 | 1:3000 | Mouse |
| β-Actin | Abclonal | AC004 | 1:100000 | Mouse |
| PI3K | Abcam | Ab191606 | 1:1000 | Rabbit |
| Phospho-PI3K (Y607) | Abcam | Ab182651 | 1:1000 | Rabbit |
| MYH7 | Abclonal | A7654 | 1:1000 | Rabbit |
| MYH2 | Abclonal | A15292 | 1:1000 | Rabbit |
| MYH4 | Proteintech | 20140-1-AP | 1:1000 | Rabbit |
| Mouse monoclonal anti-MYH I | DSHB | BA-D5 | 1:1000 | Mouse |
| Mouse monoclonal anti-MYH IIa | DSHB | SC-71 | 1:1000 | Mouse |
| Mouse monoclonal anti-MYH IIb | DSHB | BF-F3 | 1:1000 | Mouse |
| Goat anti-Mouse IgG2b | Invitrogen | A21140 | 1:400 | Goat |
| Goat anti-Mouse IgG1 | Invitrogen | A21121 | 1:400 | Goat |
| Goat anti-Mouse IgM | Invitrogen | A21044 | 1:400 | Goat |
| MYHC-1/Fast | Sigma-Aldrich | M4276 | 1:1000 | Mouse |
| MYHC-2b/Slow | Millipore | MAB1628 | 1:1000 | Mouse |
| IDH2 | Proteintech | 15932-1-AP | 1:2000 | Rabbit |
| PGC-1α | Novus | NBP1-04676 | 1:1000 | Rabbit |
| TFAM | Proteintech | 22586-1-AP | 1:1000 | Rabbit |
| Histone 3 | Abcam | Ab1791 | 1:3000 | Rabbit |
| H3K9me3 | Abcam | Ab176916 | 1:1000 (WB)  1:200 (CHIP) | Rabbit |
| H3K4me3 | Abcam | Ab8580 | 1:1000 | Rabbit |
| H3K9ac | Abmart | P37961-16f | 1:1000 | Rabbit |
| H3K9me2 | Abclonal | A2365 | 1:1000 | Rabbit |
| H3K36me3 | Abclonal | Ab9050 | 1:1000 | Rabbit |
| H3K27me3 | Abcam | Ab6002 | 1:1000 | Rabbit |
| H3K27ac | Abcam | Ab4729 | 1:1000 | Rabbit |
| Setdb2 | Abclonal | A7319 | 1:1000 | Rabbit |
| Jmjd2a | CST | 5328 | 1:1000 | Rabbit |
| Jmjd2b | CST | 8639 | 1:1000 | Rabbit |
| Jmjd2c | Novus | NBP1-49600 | 1:1000 | Rabbit |
| Jmjd2d | Abcam | Ab93694 | 1:1000 | Rabbit |
| Setdb1 | Abcam | Ab12317 | 1:1000 | Rabbit |
| HPR-conjugated Goat anti-Rabbit IgG | Abclonal | AS014 | 1:5000 | Goat |
| HPR-conjugated Goat anti-Mouse IgG | Abclonal | AS003 | 1:5000 | Goat |

**Supplementary Table 3 qPCR primer sequences**

| Gene | species | Forward | Reverse |
| --- | --- | --- | --- |
| 18S | Mouse | TTGACGGAAGGGCACCACCAG | GCACCACCACCCACGGAATCG |
| GAPDH | Mouse | GGCATGGACTGTGGTCATGA | TTCACCACCATGGAGAAGGC |
| IRS1 | Mouse | TGGACATCACAGCAGAATGAAGA | GACGTGAGGTCCTGGTTGTG |
| PI3K | Mouse | TATTGCGAGGGAAGCGAGAC | ACTTCGCCGTCTACCACTAC |
| AKT | Mouse | ACCCCCAGACCGATATGACA | CTCGGATGCTGGCTGAGTAG |
| Myh7 | Mouse | GAGTCCCAGGTCAACAAGCTG | GCTACTCCTCATTCAGGCCCTTG |
| Myh2 | Mouse | CTCGTTTGCCAGTAAGGGTC | TCCGCCACAAAGACAGATGTT |
| Myh4 | Mouse | TCATCTGGTAACACAAGAGGTGC | CTTCCGGAGGTAAGGAGCAG |
| Tnni1 | Mouse | CACAGGACACGAACAGGTGC | GCATGAGTTTACGGGAGGCA |
| Tnnc1 | Mouse | GCGGTAGAACAGTTGACAGAG | CCAGCTCCTTGGTGCTGAT |
| Tnnt1 | Mouse | AAGGGGAGCGTGTGGATTTTG | TCCTCCTTTTTCCGCTGTTCA |
| Tnni2 | Mouse | TCTCAGGATGGGAGATGAGGA | TCACACTCTTCAGGTGCTGTC |
| Tnnt2 | Mouse | CAGAGGAGGCCAACGTAGAAG | CTCCATCGGGGATCTTGGGT |
| IDH2 | Mouse | GACAGTCACCCGCCATTACC | AGCGTCTGTGCAAACCTGATA |
| PGC-1α | Mouse | AAGGTCCCCAGGCAGTAGAT | CATAGCTGTCGTACCTGGGC |
| TFAM | Mouse | GAATGTGGAGCGTGCTAAAAG | TCGGAATACAGACAAGACTGATAG |
| Tfb1m | Mouse | CGGGAGATCATTAAGTTGTTCGG | GCCCAGGACCCACTTCATAAA |
| Tfb2m | Mouse | GGCCCATCTTGCATTCTAGGG | CAGGCAACGGCTCTATATTGAAG |
| Actin | Mouse | ACTGTCGAGTCGCGTCCA | ATCCATGGCGAACTGGTGG |
| SETDB2 | Mouse | TGGGTCTGCCACAAATGGAG | TCCAGTGTTTGCGTGTTACTC |
| Ehmt1 | Mouse | GAACAGGAGTCTCCCGACAC | GGGCTGTCAGTCTTCCCTC |
| Ehmt2 | Mouse | AGCCAAGAGGGGTCTCCAAT | CTCGCTGATGCGGTCAATCT |
| Lsd1 | Mouse | ATGGATGTCACACTTCTGGA | CAAGACCTGTTACAACCATG |
| Jmjd2a | Mouse | GAGTGGCGACAACAGCAATC | GTAGGCAATGTAGCGGCTGA |
| Ezh2 | Mouse | TGCCTCCTGAATGTACTCCAA | AGGGATGTAGGAAGCAGTCATAC |
| Ash1l | Mouse | CCTCGGTGGACTAAAGTGGTG | CGCTGGCTCAGAACTATTTGA |
| SETDB1 | Mouse | CCTGGGTGCATGAGTTTGG | TGTACTGACGAAGTTCCTCCATA |
| Jmjd1a | Mouse | ACGAACACTTAGGCAAAAGCAC | GGAAGGGCTCCTCCCTTTCAA |
| Kdm5a | Mouse | AAGCAAAAACACAGGCAGAGC | TTTGGCTGATGACACACGGA |
| Phf8 | Mouse | CATGGAGTCCTAAAGCCCGTG | GGTGTCAACTCTTACCTGCTG |
| Nsd2 | Mouse | AGTACGTGTGTCAGCTGTGTG | CTTCGGGAAAGTCCAAGGCA |
| 5S rRNA | Mouse | GGCCATACCACCCTGAACGC | CAGCACCCGGTATTCCCAGG |
| 12S rRNA | Mouse | CGTTAGGTCAAGGTGTAGCCA | AATTTGAGGAGGGTGACGGG |

**Supplementary Table 4** **siRNA oligonucleotides and CHIP-qPCR primer sequences**

| si-IDH2-296-a | UACUUGAGCUGAACAUCCACG |
| --- | --- |
| si-IDH2-296-s | CGUGGAUGUUCAGCUCAAGUA |
| si-IDH2-444-a | UUUCUUCAGCUUGAACUCUUC |
| si-IDH2-444-s | GAAGAGUUCAAGCUGAAGAAA |
| si-IDH2-1337-a | UUCAGCUUCACAUUGCUGAGG |
| si-IDH2-1337-s | CCUCAGCAAUGUGAAGCUGAA |
| IDH (CHIP) F | AGGTGTAGCCTTGTTAAAGATAGTG |
| IDH (CHIP) R | ACAGTTCAGAGGTTCAGTCCAT |

**Supplementary Table 5 The coding sequence of IDH2 overexpression lentiviral vector**

| LV-shIDH2 CDS |
| --- |
| atggccggctacctgcgggctgtgagctcgctctgcagagcctcgggctcagcgcggacctgggcaccggcagcactgactgtccccagctggccggagcagccgcggcgccactatgctgagaagaggatcaaggtggagaagccggtagtggagatggacggtgacgagatgacccggatcatctggcagttcatcaaggagaagctcatcctgcctcacgtggatgttcagctcaagtattttgaccttgggcttccaaaccgtgaccagaccaatgaccaggtcaccattgactctgctctggccacccagaagtacagtgtggctgtcaagtgtgccacaatcacccctgatgaggcccgtgtggaagagttcaagctgaagaaaatgtggaagagccctaacggaacgatccggaacatccttgggggaaccgtcttcagagagccaatcatctgcaaaaacatcccccgccttgtccctggctggaccaagcccatcaccattggcaggcacgcccatggcgaccagtacaaggccacagattttgtggtagatcgagctggcacgttcaagttggtcttcaccccaaaggatggcagcagtgccaaggagtgggaggtgtataacttccctgccggaggcgttgggatggggatgtacaacaccgacgagtccatttcgggcttcgcgcacagctgcttccagtactctatccagaagaaatggccgctctacttgagcaccaagaacaccattctgaaggcctatgacgggcgtttcaaggacatcttccaggagatctttgacaagcactataagactgacttcgacaagaataagatctggtatgaacatcggctcatcgacgacatggtggcccaggtgctcaagtcttccggtggctttgtgtgggcttgcaaaaactatgatggagacgtgcagtctgacatcctggctcaaggctttggctccctcggcctgatgacatctgtgctggtctgccctgatgggaagacaattgaggctgaggctgctcatgggacagtcacccgccattaccgagaacaccagaagggccggcccaccagtaccaaccctattgccagcatctttgcctggacacggggtctggagcatcgtgggaagctggatgggaaccaggaccttatcaggtttgcacagacgctggagaaggtgtgcgtgcagactgtggagagcggagcgatgaccaaggacctggctggctgtatccatggcctcagcaatgtgaagctgaacgagcacttcctgaacaccacagacttcctggacaccattaagagcaacctggacagagctctgggcaagcagtaa |

**Supplementary References**

S1. Bonnefond A, Froguel P, Vaxillaire M. The emerging genetics of type 2 diabetes. Trends Mol Med. 2010;16:407-16.

S2. Han L, Ren C, Li L, Li X, Ge J, Wang H, et al. Embryonic defects induced by maternal obesity in mice derive from Stella insufficiency in oocytes. Nat Genet. 2018;50:432-42.

S3. Chen Y, Ma G, Gai Y, Yang Q, Liu X, de Avila JM, et al. AMPK Suppression Due to Obesity Drives Oocyte mtDNA Heteroplasmy via ATF5-POLG Axis. Adv Sci (Weinh). 2024;11:e2307480.

S4. Lane M, Zander-Fox DL, Robker RL, McPherson NO. Peri-conception parental obesity, reproductive health, and transgenerational impacts. Trends Endocrinol Metab. 2015;26:84-90.

S5. Fitz-James MH, Cavalli G. Molecular mechanisms of transgenerational epigenetic inheritance. Nat Rev Genet. 2022;23:325-41.

S6. Xavier MJ, Roman SD, Aitken RJ, Nixon B. Transgenerational inheritance: how impacts to the epigenetic and genetic information of parents affect offspring health. Hum Reprod Update. 2019;25:518-40.

S7. Burton NO, Greer EL. Multigenerational epigenetic inheritance: Transmitting information across generations. Semin Cell Dev Biol. 2022;127:121-32.
